# Tumor seeding across specialties: a systematic review

**DOI:** 10.3389/fonc.2024.1464767

**Published:** 2024-11-13

**Authors:** Pavel Kipnis, Diya Ramanathan, Richard Hoehn, Ashok R. Jethwa, Daniel W. Karakla, Bethany Rohr, Christopher M. Sutter, Jonathan R. Mark, Sobia F. Khaja, Shawn Li, Jason Thuener, Bryan T. Carroll

**Affiliations:** ^1^ Case Western Reserve University School of Medicine, Cleveland, OH, United States; ^2^ Department of Otolaryngology – Head & Neck Surgery, University of Wisconsin Hospitals and Clinics, Madison, WI, United States; ^3^ Department of Surgical Oncology, University Hospitals Medical Center, Cleveland, OH, United States; ^4^ Department of Otolaryngology – Head & Neck Surgery, University of Minnesota, Minneapolis, MN, United States; ^5^ Department of Otolaryngology - Head & Neck Surgery, Eastern Virginia Medical School, Norfolk, VA, United States; ^6^ Department of Dermatology, University Hospitals Medical Center, Cleveland, OH, United States; ^7^ Department of Vascular and Interventional Radiology, University Hospitals Medical Center, Cleveland, OH, United States; ^8^ Department of Otolaryngology – Head & Neck Surgery, University Hospitals Medical Center, Cleveland, OH, United States

**Keywords:** tumor seeding, iatrogenic, seeding, cancer, instrumentation

## Abstract

**Background:**

Understanding shared characteristics underlying reported tumor seeding episodes can reveal when tumor seeding is most likely to occur and guide clinical decision making. Our goal was to systematically review tumor seeding across specialties and determine what types of instrumentation and primary tumor histology are associated with tumor seeding.

**Methods:**

A systematic review was conducted using PubMed and Web of Science, per PRISMA guidelines. Publications ranged from 1965 to 2022, and studies with five or more reports of seeding were included. Papers were sorted by specialty and assigned a PRISMA Level of Evidence, and data analysis was conducted based on whether each paper supported the clinical significance of seeding.

**Results:**

7,165 papers were screened with 156 papers included for analysis. Overall, there were 8,161 cases of tumors seeding across specialties with the majority from general surgery, gastroenterology, and urology. Laparoscopy (n=1,561) and needle biopsy (n=3,448) were most frequently implicated, and carcinomas (n=5,778) and adenocarcinomas (n=1,090) were the most common primary tumor types.

**Discussion:**

Upon review of the most updated (2023) versions of the NCCN and NICE guidelines across all cancer types, there were identified gaps in the coverage of tumor seeding within these guidelines, with tumor seeding being entirely absent from certain guidelines and partially absent from others.

**Conclusions:**

Given the high cumulative reports of seeding and the deadly and disseminated nature of secondary disease, it is important to consider seeding risk when manipulating tumors and to modify current cancer care guidelines (NCCN/NICE) to ensure that they appropriately address seeding risk.

## Introduction

Iatrogenic tumor seeding has been defined as the movement of tumor cells from a primary tumor

to surrounding interstitial fluid, vasculature, or along the site of a surgical incision or needle tract, resulting in implantation and possible secondary tumor growth (1). Tumor manipulation carries a risk of tumor seeding which has not been previously quantified across specialties. The factors that underly iatrogenic tumor seeding are poorly understood. Previous studies have cited degree of primary neoplastic differentiation (2), lymph node metastasis (3), primary tumor histology (4), and type of surgical procedure (5) as risk factors in iatrogenic seeding, but no consensus exists regarding “high risk” tumor types and instrumentation (6).

Oncologic treatment is primarily guided by adherence to evidence-based guidelines, namely the National Comprehensive Cancer Network (NCCN) guidelines in the United States and the National Institute for Health and Care Excellence (NICE) guidelines in the United Kingdom. These guidelines should adequately encompass the existing literature on tumor seeding to guide clinical decision-making in cancer treatment.

The aim of this study was to systematically review tumor seeding across all specialties and determine what types of instrumentation and primary tumor histology are associated with seeding. Secondary aims include exploring trends across tumor stage, tumor grade, and duration between tumor manipulation and seeding, as well as trends in reports across the decades. Understanding the commonalities that seeded tumors share should guide the revision of current NCCN recommendations to provide specific guidelines for seeding prevention.

## Methods

### Literature search

A systematic review using the Preferred Reporting Items for Systematic Reviews and Meta-Analysis (PRISMA-P) statement and registered at the Prospective Register of Systematic Reviews (PROSPERO) (7) was conducted using PubMed and Web of Science on June 28, 2022. Publications were not restricted by date and ranged from 1965 to 2022. Search criteria were developed with a reference librarian (**Appendix 1**). Duplicate references were excluded, and PRISMA reporting guidelines were followed (8). Seeding was defined as the transplantation of tumor cells or secondary tumor growth at a site distinct from the primary tumor with confirmed shared origins.

### Inclusion and exclusion criteria

Title, abstract, and full-text screens were conducted independently by two researchers. For the title screen, exclusion criteria were as follows: non-English, non-human, letters, and non-seeding publications.

Non-seeding publications were defined as those without the terms “seeding”, “contamination”, “rupture”, “spillage”, “implantation”, “disseminated”/”dissemination”, or “complication” OR publications with any type of instrumentation in the title along with one of the following: “metastasis”, “spread”, “extension”, “residue”, “recurrence”, “dislodge”, “displace”, “morcellation”, or “cancer risk”.

The abstract screen excluded non-English and non-human publications as well as letters, comments, and case series or cohort studies with fewer than five patients based on precedent from previous tumor seeding systematic reviews. In the manuscript screen, papers were excluded if they were non-English, non-human/*in vitro* studies, editorial papers describing findings from a paper or papers that were already included, contained fewer than five cases of tumor seeding, or described treatment options following seeding rather than the characteristics of seeded tumors.

Following the full-text screen, qualitative systematic reviews that cited other papers that provided

quantitative tumor seeding data (>5 patients) were noted and included in the final subset of accepted papers. The PRISMA Levels of Evidence (9) system was employed for each included paper (**Appendix 2**).

### Inter-rater agreement

All rounds of screening were performed independently by two investigators (DR & PK); discrepancies were reconciled to reach consensus. Prior to reconciliation, the values for percentage agreement were 98.9% for the title screen, 96.8% for the abstract screen, and 100.0% for the full-text screen, with Cohen’s kappa values of 0.96, 0.89, and 1.00 respectively. The process of reconciliation yielded 100% agreement (Cohen’s kappa = 1.00) for all screens (10).

### Data extraction

Papers were sorted based on medical specialty and evaluated based on whether they supported the biological existence of seeding and/or the clinical significance of seeding. Papers that supported the biological existence of seeding were those that validated tumor seeding as a complication of tumor manipulation, and papers that supported the clinical significance of seeding were those that either endorsed higher rates of tumor seeding with a specific instrumentation or proposed changes to clinical practice based on the findings. Data was analyzed by specific instrumentation type (**Table 1**), with a grouped analysis based on instrumentation (**Table 2**), as well as tumor histology (**Table 3**).

**Table 1:** Reports of seeding stratified by instrumentation across specialties.

			Paper level of evidence						Conclusions on seeding (n=)	
Instrumentation	Total papers	Cases of associated seeding	1	2	3	4	5	6	Paper supports biological existence	Paper supports clinical importance
Dermatology										
Percutaneous needle biopsy	1	28	0	0	0	1	0	0	1 (n=28)	1 (n=28)
Gastroenterology										
Catheter procedures	2	39	0	0	0	2	0	0	2 (n=39)	1 (n=23)
Laparoscopy	6	330	0	0	0	3	2	1	6 (n=330)	3 (n=229)
General Surgery										
Catheter procedures	2	38	0	0	0	1	1	0	2 (n=38)	2 (n=38)
Laparoscopy	23	869	1	0	0	11	8	3	23 (n=869)	9 (n=492)
Probe procedures	3	79	0	0	0	3	0	0	3 (n=79)	3 (n=79)
Open surgery	8	57	0	0	1	5	0	2	8 (n=57)	6 (n=38)
Gynecologic Oncology										
Dilation & curettage (D&C)	1	49	0	0	0	0	1	0	1 (n=49)	0 (n=0)
Episiotomy	1	18	0	0	0	0	1	0	1 (n=18)	1 (n=18)
Hysteroscopy	2	69	0	0	0	1	1	0	2 (n=69)	1 (n=10)
Laparoscopy	10	257	0	0	0	2	6	2	9 (n=245)	6 (n=107)
Morcellation	2	36	0	0	0	1	1	0	2 (n=36)	2 (n=36)
Saline infusion sonohysterogram (SIS)	1	17	0	0	0	0	1	0	1 (n=17)	0 (n=0)
Interventional Radiology										
Needle biopsy	70	3448	1	0	1	49	12	7	67 (n=3372)	26 (n=1292)
Orthopedic surgery										
Open biopsy	1	20	0	0	0	1	0	0	1 (n=20)	1 (n=20)
Otolaryngology – Head and Neck Surgery										
PEG tube placement	12	459	0	0	0	3	7	2	12 (n=459)	7 (n=207)
Skull base surgery^2^	3	114	0	0	0	0	3	0	3 (n=114)	2 (n=100)
Thoracic surgery										
Pleural procedures^1^	3	95	0	0	0	2	1	0	3 (n=106)	2 (n=83)
Video-assisted thoracoscopic surgery (VATS)	3	38	0	0	0	1	1	1	3 (n=38)	0 (n=0)
Urology										
Laparoscopy	5	105	0	0	0	1	4	0	5 (n=105)	1 (n=25)
Ureteroscopy	4	2024	0	0	0	3	1	0	4 (n=2024)	4 (n=2024)

^1^Pleural procedures include intercostal tube drainage, thoracentesis, pleural biopsy, pleural thoracoscopy/thoracotomy, and chest tube placement.

^2^Skull base surgery includes craniotomy and transseptal/transfacial/transpalatal interventions.

**Table 2:** Pooled reports of seeding grouped by instrumental paradigm.

			Paper level of evidence						Conclusions on seeding (n=)	
Instrumentation	Total papers	Cases of associated seeding	1	2	3	4	5	6	Paper supports biological existence	Paper supports clinical importance
All specialties										
Needle biopsy	70	3448	1	0	1	49	12	7	67 (n=3372)	26 (n=1292)
Open surgery	12	286	0	0	1	6	3	2	12 (n=297)	8 (n=241)
Minimally invasive surgery*	68	3968	1	0	0	31	29	7	68 (n=3968)	32 (n=3081)
PEG tube placement	12	459	0	0	0	3	7	2	12 (n=459)	7 (n=207)

*Includes laparoscopy, endoscopy, catheter, probe, etc.

**Table 3:** Reports of seeding stratified by histology across specialties.

			Level of Evidence (n=)						Conclusions on seeding (n=)		
Cytology of Primary Tumor	Total papers (n=)	Cases of associated seeding (n=)	1	2	3	4	5	6	Paper supports biological existence	Paper supports clinical importance	Mean duration until seeding (mo)
Dermatology											
Melanoma	1	28	0	0	0	1	0	0	1 (n=4)	1 (n=4)	11.6
Gastroenterology											
Adenocarcinoma	5	75	0	0	0	4	1	0	5 (n=75)	1 (n=11)	16.7
Carcinoma	19	1117	0	0	0	12	5	2	19 (n=1117)	10 (n=932)	13.9
General Surgery											
Adenocarcinoma	10	780	1	0	0	2	3	4	10 (n=780)	6 (n=198)	10.6
Breast cancer (unspecified)	1	15	0	0	0	1	0	0	1 (n=15)	0 (n=0)	29.7
Carcinoma	33	1969	0	0	1	22	5	5	33 (n=1969)	22 (n=580)	13.9
Colorectal cancer (unspecified)	12	282	1	0	1	6	3	1	12 (n=282)	6 (n=126)	20.0
Gallbladder cancer (unspecified)	2	24	0	0	0	0	2	0	2 (n=24)	1 (n=12)	11.6
Gastric cancer (unspecified)	5	113	0	0	0	4	1	0	5 (n=113)	2 (n=2)	9.7
Gastrointestinal cancer (unspecified)	1	8	0	0	0	0	0	1	1 (n=8)	1 (n=8)	4.8
Hepatic cancer (unspecified)	1	2	0	0	0	0	1	0	1 (n=2)	1 (n=2)	18.4
Liposarcoma	1	5	0	0	0	1	0	0	1 (n=5)	0 (n=0)	45.0
Melanoma	1	2	0	0	0	1	0	0	1 (n=2)	1 (n=2)	3.0
Osteosarcoma	1	11	0	0	0	1	0	0	1 (n=11)	1 (n=11)	38.2
Pancreatic cancer (unspecified)	2	35	0	0	0	1	1	0	2 (n=35)	1 (n=1)	18.7
Gynecologic Oncology											
Adenocarcinoma	6	153	0	0	0	1	4	1	6 (n=153)	3 (n=13)	12.7
Carcinoma	8	107	0	0	0	3	4	1	8 (n=107)	6 (n=103)	9.3
Leiomyosarcoma	1	39	0	0	0	0	1	0	1 (n=39)	1 (n=39)	N/A
Ovarian cancer, endometrial cancer, cervical cancer* (unspecified)	2	27	0	0	0	0	2	0	2 (n=27)	1 (n=20)	9.6
Uterine mesenchymal neoplasms (unspecified)	1	12	0	0	0	1	0	0	1 (n=12)	1 (n=12)	7.2
Ophthalmology											
Melanoma	1	8	0	0	0	0	0	1	1 (n=8)	0 (n=0)	N/A
Orthopedic Surgery											
Sarcoma (bone/soft tissue)*	2	29	0	0	0	1	1	0	2 (n=29)	1 (n=21)	45
Otolaryngology – Head and Neck Surgery											
Adenocarcinoma	5	8	0	0	0	0	2	3	5 (n=8)	4 (n=6)	13.0
Carcinoma	14	452	0	0	0	2	8	4	14 (n=452)	8 (n=192)	24.1
Carcinosarcoma	1	4	0	0	0	0	1	0	1 (n=4)	0 (n=0)	7.0
Chordoma	3	71	0	0	0	0	3	0	3 (n=71)	2 (n=57)	33.1
Craniopharyngioma	1	36	0	0	0	0	1	0	1 (n=36)	1 (n=36)	36.0
Head and neck cancer (unspecified)	2	36	0	0	0	2	0	0	2 (n=36)	1 (n=31)	26.0
Metastatic melanoma, pleomorphic adenoma, adenocarcinoma, squamous cell carcinoma*	1	7	0	0	0	0	1	0	1 (n=7)	0 (n=0)	19.5
Thoracic Surgery											
Adenocarcinoma	2	16	0	0	0	1	0	1	2 (n=16)	1 (n=10)	16.4
Lung cancer (unspecified)	4	90	0	0	0	3	1	0	4 (n=90)	1 (n=6)	19.3
Metastases to the lung	1	6	0	0	0	0	0	1	1 (n=6)	0 (n=0)	7.6
Non-small cell lung cancer (NSCLC)	4	294	0	0	0	4	0	0	3 (n=226)	1 (n=191)	32.0
Pleural mesothelioma	3	67	0	0	0	2	0	1	3 (n=67)	1 (n=46)	8.4
Small cell lung cancer	1	1	0	0	0	0	0	1	1 (n=1)	0 (n=0)	7.6
Squamous cell carcinoma & adenocarcinoma	7	128	0	0	0	4	2	1	3 (n=128)	3 (n=65)	21.4
Urology											
Adenocarcinoma	3	58	0	0	0	1	2	0	3 (n=58)	2 (n=16)	18.6
Carcinoma	12	2133	0	0	0	5	6	1	12 (n=2133)	11 (n=1080)	15.8
Germ cell tumor	1	2	0	0	0	0	1	0	1 (n=2)	1 (n=2)	N/A
Leiomyosarcoma	1	1	0	0	0	0	1	0	1 (n=1)	1 (n=1)	12.9
Prostate cancer (unspecified)	2	6	0	0	0	1	1	0	2 (n=6)	2 (n=6)	16.0

*Grouped together in primary source. NA, Not applicable.

Secondary analysis of tumor stage, tumor grade, and duration till seeding was conducted for papers that reported on these variables. These three variables were defined categorically as follows: low-stage (stage I-II or equivalent) vs. high-stage (stage III-IV or equivalent), low-grade vs. intermediate-grade vs. high-grade, and short duration (<6mo) vs. long duration (>6mo). Sampled t-tests (for tumor staging and duration) and ANOVA tests (for tumor grading) were performed for nominal variables to assess for statistically significant variations (p<0.05). Additionally, papers were organized by year of publication and sorted by conclusion for each decade. Due to data heterogeneity, overall seeding incidence across specialties was not calculated.

### NCCN and NICE guideline review

The most updated NCCN and NICE guidelines were referenced to evaluate current recommendations. Two investigators (DR & PK) independently reviewed each guideline.

## Results

**Figure 1:**
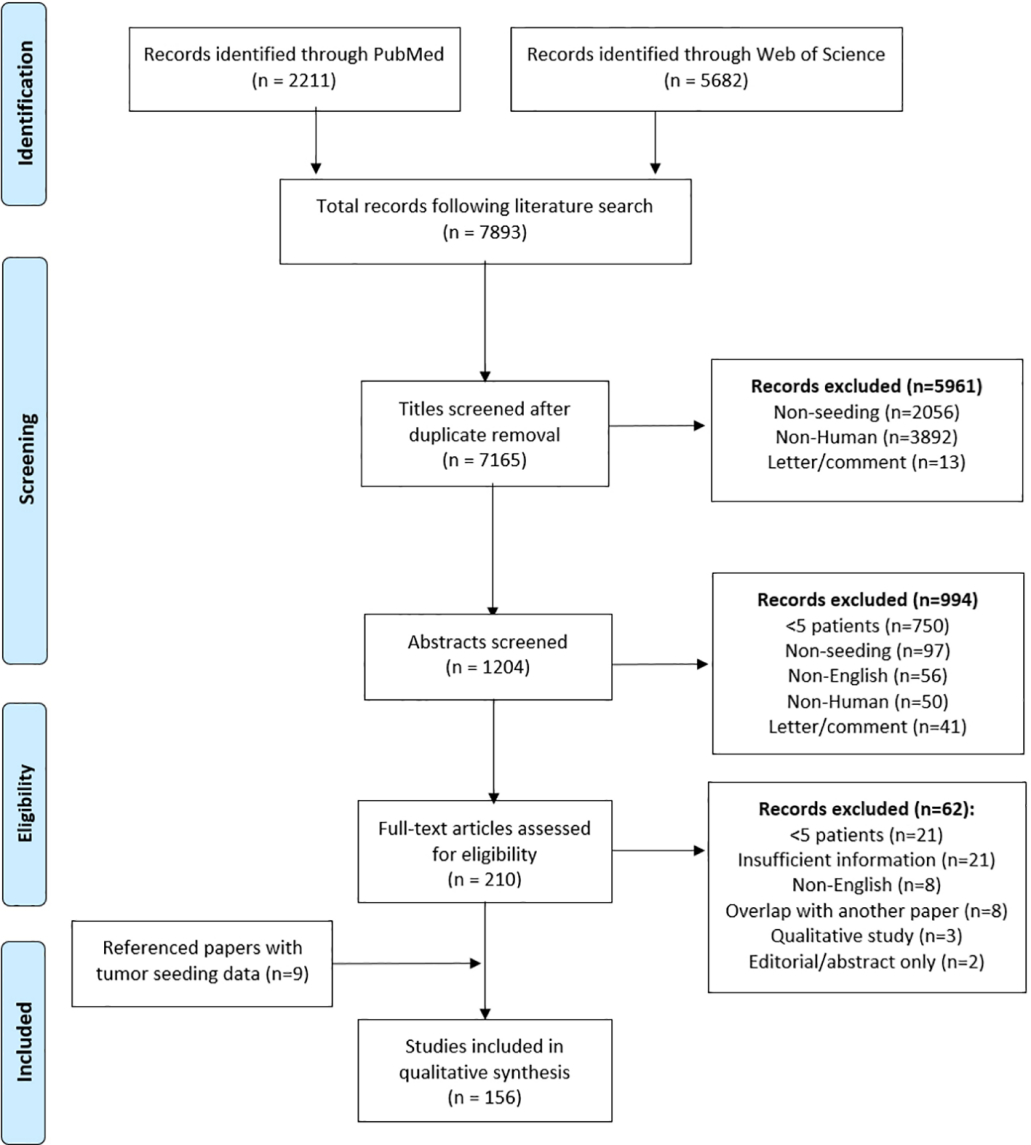
PRISMA Flow Diagram for Tumor Seeding.

The search yielded 7,893 results, representing 7,165 papers after duplicates were removed. After

title, abstract, and full-text screens, 147 papers remained. Three papers provided a qualitative review of a total of nine referenced papers, citing quantitative tumor seeding data, and these papers were included resulting in a total of 156 papers in the final analysis (**Figure 1**). Of these papers, 22 were in gastroenterology, 60 general surgery, 17 gynecologic oncology, 19 otolaryngology, 18 thoracic surgery, 16 urology, 1 dermatology, 1 ophthalmology, and 2 orthopedic surgery. Discrepancies between number of cases in the instrumentation and histology tables were attributable to variable or incomplete data reporting across included papers. All needle biopsy procedures were classified as interventional radiology (IR) as these procedures are performed through IR consultation.

In gastroenterology, catheter procedures (n=39) and laparoscopy (n=330) were associated with tumor seeding, with Nine out of 22 papers (n=252) supporting the clinical importance of seeding (**Supplementary Table 3**). By histology, the associated tumor types were adenocarcinoma (n=75) and carcinoma (n=1117), with 11 papers (n=943) supporting the clinical importance of seeding (**Table 3**).

In general surgery, catheter procedures (n=38), laparoscopy (n=869), probe procedures (n=79), and open surgery (n=57) were associated with tumor seeding, with a Thirty three out of 60 papers (n=647) reinforcing the clinical importance of seeding (Table 6). Multiple tumor types were associated with seeding, the most common being adenocarcinoma (n=780) and carcinoma (n=1969) (**Table 3**).

In gynecologic oncology, there were 17 papers (n=446) with tumor seeding following dilation and curettage (D&C) (n=49), episiotomy (n=18), hysteroscopy (n=69), laparoscopy (n=257), morcellation (n=36), and saline infusion sonohysterogram (SIS) (n=17). Ten out of 17 papers (n=171) supported the clinical importance of seeding (Table 6). By histology, the commonly implicated tumor types were adenocarcinoma (n=153) and carcinoma (n=89) (**Table 3**).

Across specialties, there were 70 papers (n=3448) that reported tumor seeding following needle biopsy, categorized under interventional radiology. These cases were from gastroenterology (n=677), general surgery (n=2032), otolaryngology (n=26), thoracic surgery (n=424), urology (n=92), dermatology (n=28), ophthalmology (n=8), and orthopedic surgery (n=9).

In otolaryngology, tumor seeding was reported in 15 papers (n=573) following PEG tube placement (n=459) and skull base surgery (n=114). Nine out of 15 papers (n=307) supported the clinical importance of seeding (Table 6). Commonly implicated tumor types included carcinoma (n=452), chordoma (n=71), and craniopharyngioma (n=36) (**Table 3**).

In thoracic surgery, tumor seeding was reported in 6 papers (n=133) following pleural procedures (n=95) and video-assisted thoracoscopic surgery (VATS) (n=38). Seven out of 18 papers (n=83) supported the clinical importance of seeding (Table 6). Commonly associated tumor types included non-small cell lung cancer (NSCLC) (n=294), pleural mesothelioma (n=67), and squamous cell carcinoma/adenocarcinoma/other carcinomas (n=128) (**Table 3**).

In urology, tumor seeding was reported in 9 papers (n=2129) following laparoscopy (n=105) and ureteroscopy (n=2024). Eleven out of 16 papers (n=2049) supported the clinical importance of seeding, (Table 6). By histology, commonly implicated tumor types among papers supporting the biological existence of seeding were adenocarcinoma (n=58) and carcinoma including renal cell, transitional cell, and urothelial (2133).

In dermatology, one paper (n=28) reported melanoma-associated seeding. In ophthalmology, one paper (n=8) reported iatrogenic tumor seeding after FNA. In orthopedic surgery, tumor seeding was reported following needle biopsy and open biopsy (n=29) of sarcomas across two papers (**Tables 1**, **3**).

**Figure 2:**
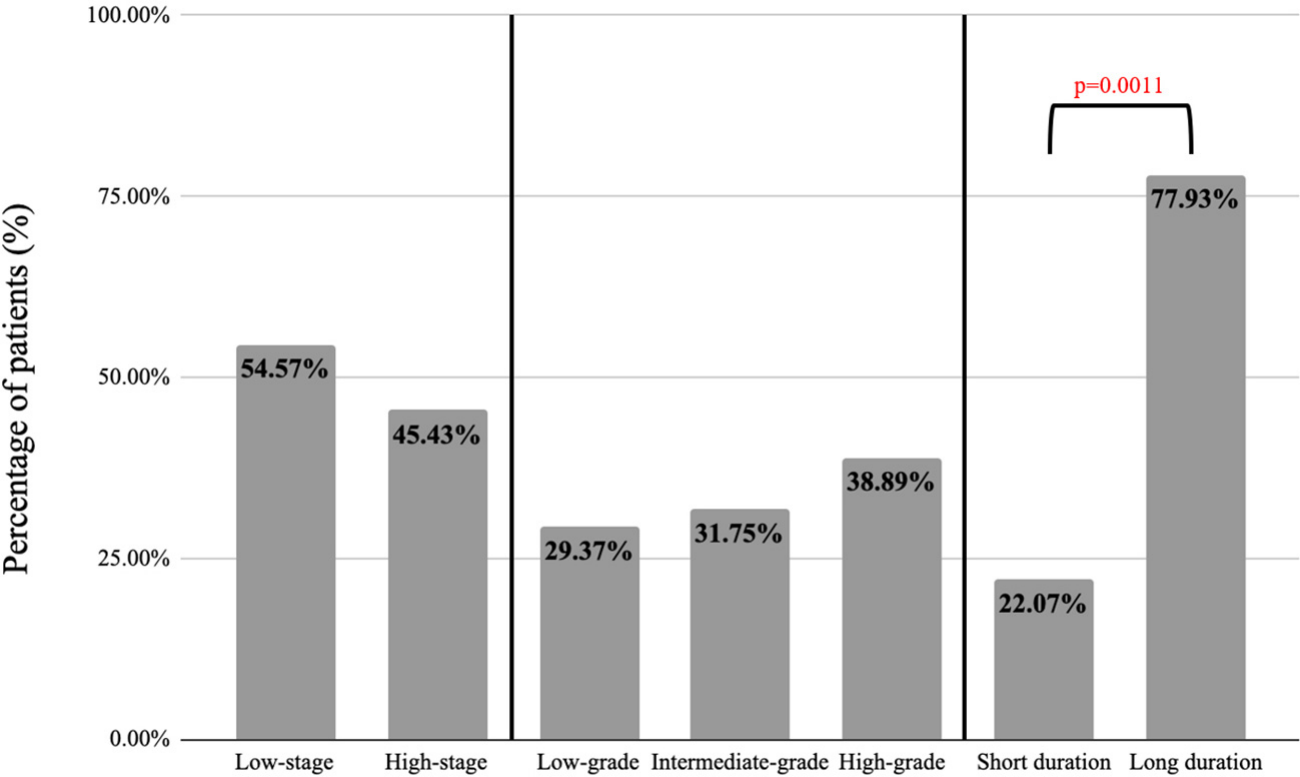
Stage, grade, and duration until seeding for seeded tumors.

61 papers (n=1730) incorporated information on tumor staging, 54.57% low-stage tumors and 45.43% high-stage tumors (p=0.42). For tumor grade, there were 11 papers (n=126), with 37 patients (29.37%) having low-grade tumors, 40 patients (31.75%) with intermediate-grade, and 49 patients (38.89%) with high-grade (p=0.87). Of the reported cases of seeding, 4,491 cases described duration until presentation of secondary tumor, with 22.07% reported as short-duration (within 6 months of intervention) and 77.93% long-duration (more than 6 months after intervention) (p=0.0011) (**Figure 2**).

**Figure 3:**
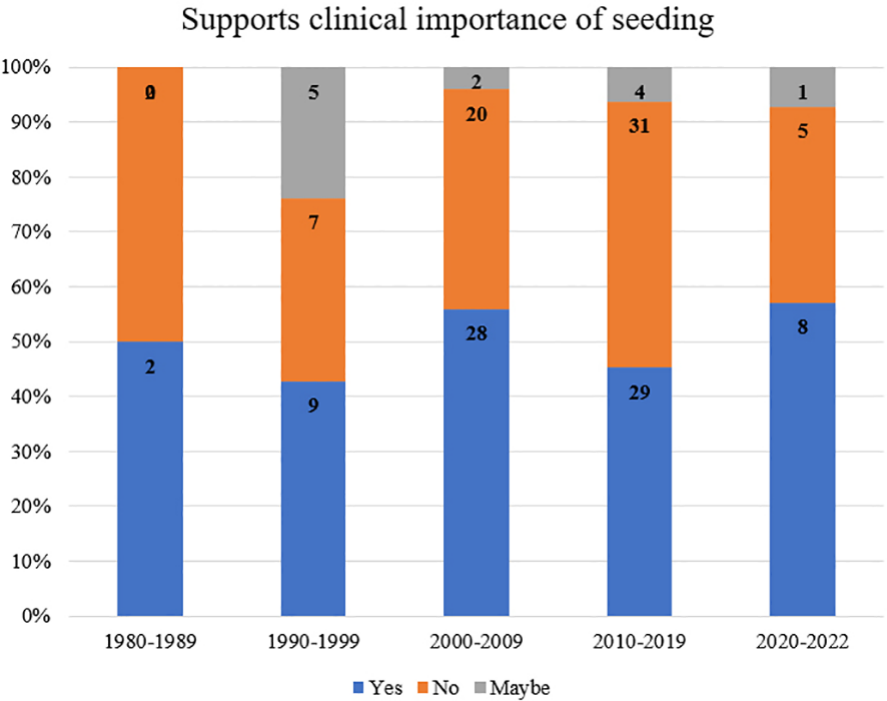
Reports of seeding across decades.

When analyzed by year of publication and stratified by support of clinical importance of seeding, 50% of papers from 1980-1989 supported the clinical importance of seeding, 43% from 1990-1999, 56% from 2000-2009, 48% from 2010-2019, and 57% from 2020-2022 (**Figure 3**).

## Discussion

Iatrogenic tumor seeding is a potentially serious complication that occurs when cancer cells are inadvertently spread to another location during a surgical procedure. Seeding can have catastrophic consequences, especially for patients who would otherwise have a favorable long-term prognosis (11). This study describes a high total number of reported cases of seeding (n=8,161) across specialties, with most reports coming from general surgery, gastroenterology, and urology. When evaluated by instrumentation, minimally invasive surgery (n=3968) and needle biopsy (n=3448) were most frequently implicated in iatrogenic seeding, with many reports describing metastasis along the needle tract or port site. Histological reports of primary tumors across specialties demonstrate that the most common tumors associated with seeding were carcinomas (n=5778) and adenocarcinomas (n=1090) across organ systems. Notably, poor differentiation has also been associated with increased propensity for tumor seeding in carcinoma (2). While iatrogenic tumor seeding is a rare occurrence, physicians should be aware of the risk associated with various instrumental techniques and tumor types.

Most reports of seeding were in patients who underwent percutaneous needle biopsy or laparoscopy. Although this is may be a function of the frequency of tumor manipulation with these techniques, many reports in this study directly attributed the cases of tumor seeding to needle biopsy (12–14) or laparoscopy (15, 16). In gastroenterology, general surgery, and thoracic surgery, needle biopsy was most associated with clinically significant cases of seeding. In gynecologic oncology, laparoscopy was most associated with clinically significant cases of seeding, and laparoscopy was the second most common cause of clinically significant seeding in gastroenterology and general surgery. Notably, PEG tube placement accounted for 459 cases of seeding out of a total of 599 reports in the ENT literature.

The location of the tumor being manipulated is relevant because this determines the depth of normal tissue parenchyma that is penetrated and may underlie risk of seeding associated with techniques like needle biopsy. This suggests that evaluating the risks of iatrogenic seeding should be performed on case-by-case basis, with focus placed not only on tumor size, location, and histology, but also future patient disease management (17, 18). The impact of tumor seeding on patient prognosis supports the adoption of prophylactic measures in high-risk patients (19–22).

Some specialties have recognized the risks associated with undiagnosed lesion biopsy; hepatobiliary surgeons advocate for non-invasive imaging and serological tumor markers for diagnosis due to poorer long-term survival after biopsy of solid lesions. However, in the case of suspected sarcomas, tumors are best evaluated with core needle biopsy following initial appropriate imaging assessment. In this study, most cases of seeding were associated with carcinoma or adenocarcinoma, with the exception of melanoma being associated with clinically significant seeding in dermatology and non-small cell lung cancer being most associated with pleural recurrence in thoracic surgery (23, 24).

Generally, there appears to be a predilection for seeding among tumors of epithelial origin. Previous studies evaluating histologic characteristics of seeded tumors have shown that poorly differentiated tumors and invasive carcinomas were associated with higher rates of seeding (2, 4, 25, 26) and earlier recurrence (27–30). Additionally, lymph node metastasis is a reported risk factor for seeding in patients with hepatocellular carcinoma (3). Taken together, these data further support that invasive diagnostic procedures should be evaluated on a case-by-case basis, taking into account likely origins of a malignancy and associated risks.

Upon review of the most updated (2023) versions of the NCCN guidelines across all cancer types, tumor seeding was found to be referenced in the following NCCN guidelines: bladder cancer, bone cancer, gastric cancer, gastrointestinal stromal tumors, hepatocellular carcinoma, uveal melanoma, pleural mesothelioma, pancreatic adenocarcinoma, soft tissue sarcoma, thymoma/thymic cancer, and Wilms tumor. Notably, tumor seeding was entirely absent from the NCCN guidelines for the following surgical specialties in our review: dermatology, gynecologic oncology, and otolaryngology. Tumor seeding was also partially absent from the NCCN guidelines for the following surgical specialties: gastroenterology, general surgery, thoracic surgery, and urology. The NICE guidelines referenced tumor seeding in the context of liver cancer, rectal cancer, lung cancer surgical metastasis, and breast cancer, with all guidelines discussing the hypothetical risk of seeding without specific guidelines for prophylaxis or treatment. Given these findings, we recommend the revision of these guidelines to include the reported risk of tumor seeding and associated appropriate prophylactic measures.

When stratified by tumor stage and tumor grade, there were similar rates of tumor seeding between low-stage and high-stage tumors and across tumor grade (**Figure 2**). Previous studies have endorsed a relationship between advanced stage tumors and iatrogenic seeding following laparoscopy across colorectal, gallbladder, gastric, hepatic, pancreatic, ovarian, endometrial, and cervical cancers (31–37), as well as following PEG tube placement in head and neck tumors (**Appendix 2**). Previous publications on tumor seeding have reported a relationship between high grade tumors and iatrogenic seeding, namely following laparoscopy for renal cell carcinomas and transitional cell carcinomas, percutaneous radiofrequency ablation of hepatocellular carcinoma, and PEG tube placement for head and neck adenocarcinomas (**Appendix 2**) (**Appendix 3**). Our data reveal that across tumor types, there is no clear relationship between tumor staging or grading and seeding, although specific tumor types may have a propensity for seeding at advanced stages and grades.

Among papers that reported duration until seeding, the data showed that patients with iatrogenic seeding were significantly more likely to present >6mo after their initial intervention (18, 22, 38–46) (**Figure 2**). These results can inform future surveillance for tumors or procedures with a high risk of seeding, which may be most useful >6mo after the primary procedure. When stratified by decade of publication, publications from 1980-2022 consistently supported the biological existence of seeding, with rates of 97-100% of papers in support across five decades. Rates of support of the clinical importance of seeding ranged from 43% (1990-1999) to 57% (2020-2022), supporting heterogeneous conclusions on the clinical importance of tumor seeding over time (**Figure 3**). The highest proportion of equivocal publications occurred from 1990-1999, indicating a tendency towards ambiguous language in earlier publications.

Despite the methodological rigor of this systematic review, one of its limitations was the inclusion

of almost entirely retrospective data. Because systematic review quality is limited by existing publications, the relative absence of prospective data (47–49) and randomized controlled studies, due to the rarity of tumor seeding, is notable. When considering iatrogenic seeding, it is crucial to differentiate between true tumor seeding and local recurrence (the development of metastatic tumor proximal to the initial tumor from residual primary tumor cells) (50). Differentiating between these phenomena can be difficult, and this conflation may underlie underreported tumor seeding across specialties and confound interpretation. Finally, while our search phrase was created with a reference librarian, it is possible that there were missing publications in our final dataset, given the heterogeneous language used to describe tumor seeding.

## Conclusions

While seeding reports were seemingly rare, a cumulative analysis across specialties revealed a high total number of iatrogenic seeding cases and severe impacts on longitudinal patient outcomes, complicating otherwise promising prognoses. Acknowledging tumor types and instrumentation paradigms associated with seeding, namely carcinomas and adenocarcinomas, as well as needle biopsy and laparoscopy, can help to guide clinical decision-making regarding tumor manipulation. Specifically, modifying clinical guidelines, such as the NCCN and NICE guidelines that largely guide international oncologic decision making, is vital. Future directions include analyzing effective seeding prophylaxis and exploring elements of tumor microenvironment that allow seeding.

## Data Availability

The original contributions presented in the study are included in the article/**Supplementary Material**. Further inquiries can be directed to the corresponding author.
