# Supplementary material for: Tumor seeding across specialties: a systematic review
Source: Front Oncol. 2024 Nov 13;14:1464767. doi: 10.3389/fonc.2024.1464767 (PMC11598697; doi:10.3389/fonc.2024.1464767)
Supplement: Supplementary file 1 [file DataSheet1.docx]

**Supplemental Appendix 1: Search Strategy**

(("Neoplasm Seeding"[Mesh]) OR (seeding[tiab]) OR (rupture[tiab]) OR (contamination[tiab]) OR (spillage[tiab])) AND (("Endoscopy"[Mesh]) OR (“endoscop*”[tiab]) OR (“scop*”[tiab]) OR (“needle”[tiab]) OR ("Biopsy, Needle"[Mesh]))
