# Supplementary material for: Tumor seeding across specialties: a systematic review
Source: Front Oncol. 2024 Nov 13;14:1464767. doi: 10.3389/fonc.2024.1464767 (PMC11598697; doi:10.3389/fonc.2024.1464767)
Supplement: Supplementary file 2 [file DataSheet2.docx]

| **Gastroenterology** | | | | | | | |
| --- | --- | --- | --- | --- | --- | --- | --- |
| **Tumor cytology** | **Title** | **LOE** | **Seeding (n=)** | **Staging (n=)** | **Location of seeded tumor** | **Cytology-related risk?** | **Total (n=)** |
| Adenocarcinoma | Needle Tract Seeding: An Overlooked Rare Complication of Endoscopic Ultrasound-Guided Fine-Needle Aspiration** | 5 | 11 | T1 (2), T2 (2), T3 (3), 4 unreported | N/A | N/A | **75** |
|  | Risk of gastric or peritoneal recurrence, and long-term outcomes, following pancreatic cancer resection with preoperative endosonographically guided fine needle aspiration | 4 | 19 | IA (2), IB (2), IIA (2), IIB (1) | stomach wall (3), peritoneum (16) | N/A |  |
|  | Non-negligible rate of needle tract seeding after endoscopic ultrasound-guided fine-needle aspiration for patients undergoing distal pancreatectomy for pancreatic cancer | 4 | 6 | IA (2), IB (2), IIA (2) | needle tract | N/A |  |
|  | Incidence of port site recurrence after laparoscopic cholecystectomy for preoperatively unsuspected gallbladder carcinoma | 4 | 37 | T1b (1), T2 (2), T3 (2) | port site | N/A |  |
|  | CT of abdominal wall implantation metastases after abdominal percutaneous procedures** | 4 | 2 | I (1), IV (1) | port site | N/A |  |
| Carcinoma | Analysis of patients with tumor seeding after percutaneous radiofrequency ablation of hepatocellular carcinoma | 4 | 6 | N/A | abdominal wall (4), thoracic wall (2), douglas pouch (1) | Poorly differentiated tumors appear to have a higher risk of seeding than well-differentiated | **1117** |
|  | Needle track seeding after percutaneous radiofrequency ablation of hepatocellular carcinoma: 14-year experience at a single centre | 4 | 12 | "poorly" (6), "moderately" (4), "well" (2) - differentation of HCC | needle track | Size of the tumor (>3cm) is associated with greater risk of seeding |  |
|  | Port site metastases from gallbladder cancer after laparoscopic cholecystectomy. Results of a Swedish survey and review of published reports | 4 | 9 | N/A | Port side (abdominal wall) | N/A |  |
|  | Port site recurrences after laparoscopic cholecystectomy | 5 | 174 | T1 (10), T2 (26), "carcinoma infiltrated galllbladder wall" (20), remaining unspecified | Port site | N/A |  |
|  | Port-site metastasis following laparoscopic cholecystectomy: a review of the literature and a case report | 6 | 46 | pT3 (1), unspecified (45) | port site (case report) | N/A |  |
|  | Port site metastasis after diagnostic laparoscopy for upper gastrointestinal tract malignancies: an uncommon entity | 4 | 13 | N/A | port site or incision site | N/A |  |
|  | Needle track seeding following biopsy of liver lesions in the diagnosis of hepatocellular cancer: a systematic review and meta-analysis | 5 | 26 | N/A | needle track | N/A |  |
|  | Seeding Risk Following Percutaneous Approach to Hepatocellular Carcinoma - Seeding following percutaneous diagnostic and therapeutic approaches for hepatocellular carcinoma. What is the risk and the outcome? Seeding risk for percutaneous approach of HCC | 6 | 179 | N/A | Chest wall, subcutaneous at port site | N/A |  |
|  | Rate of seeding with biopsies and ablations of hepatocellular carcinoma: A retrospective cohort study | 4 | 6 | N/A | port site, diaphragm | N/A |  |
|  | Incidence of needle tract seeding and responses of soft tissue metastasis by hepatocellular carcinoma postradiotherapy | 4 | 39 | Well (1), Moderate (12), Poor (3) | needle tract | N/A |  |
|  | Tumour seeding after percutaneous cryoablation for hepatocellular carcinoma | 4 | 10 | "poor" (3), "moderate" (2), "well" (1) - level of differentiation of tumor | needle tract | N/A |  |
|  | Needle track seeding after percutaneous microwave ablation of malignant liver tumors under ultrasound guidance: analysis of 14-year experience with 1462 patients at a single center | 4 | 11 | N/A | needle tract, peritoneum, intercostal muscle | N/A |  |
|  | Dissemination metastasis after laparoscopic colorectal surgery versus conventional open surgery for colorectal cancer: a metanalysis | 5 | 51 | N/A | port site | N/A |  |
|  | Needle track seeding in colorectal carcinoma after local ablation by high-dose-rate brachytherapy: a retrospective study of 1,107 catheter placements | 4 | 16 | G1 (2.2%), G2 (62.3%), G3 (14.5%), remaining had no info | Needle tract | N/A |  |
|  | Needle Tract Seeding: An Overlooked Rare Complication of Endoscopic Ultrasound-Guided Fine-Needle Aspiration** | 5 | 2 | T2 (2) | N/A | N/A |  |
|  | CT of abdominal wall implantation metastases after abdominal percutaneous procedures | 4 | 4 | I (1), II (3), IV (1) | port site | N/A |  |
|  | Percutaneous transhepatic biliary drainage catheter tract recurrence in cholangiocarcinoma | 4 | 23 | T1 or T2 (12), T3 or T4 (11) | chest wall, abdominal wall, hepatic parenchyma | Papillary tumor type appears to have a higher risk of recurrence |  |
|  | A systematic review of the comparison of the incidence of seeding metastasis between endoscopic biliary drainage and percutaneous transhepatic biliary drainage for resectable malignant biliary obstruction | 5 | 483 | N/A | N/A | N/A |  |
|  | Percutaneous Preoperative Biliary Drainage for Resectable Perihilar Cholangiocarcinoma: No Association with Survival and No Increase in Seeding Metastases | 4 | 7 | N/A | abdominal wall (port site) | N/A |  |

| **General Surgery** | | | | | | | |
| --- | --- | --- | --- | --- | --- | --- | --- |
| **Tumor cytology** | **Title** | **LOE** | **Seeding (n=)** | **Staging (n=)** | **Location of seeded tumor** | **Cytology-related risk?** | **Total (n=)** |
| Adenocarcinoma | Preoperative Endoscopic Ultrasound Fine Needle Aspiration Versus Upfront Surgery in Resectable Pancreatic Cancer: A Systematic Review and Meta-analysis of Clinical Outcomes Including Survival and Risk of Tumor Recurrence | 1 | 438 | Well-differentiated (103 EUS-FNA, 228 non EUS-FNA), Moderately-differentiated (411 EUS-FNA, 885 non EUS-FNA), Poorly-differentiated (206 EUS-FNA, 610 non EUS-FNA) | Peritoneum | N/A | **780** |
|  | Analysis of circulating cell-free DNA after endoscopic ultrasound-guided fine needle aspiration in pancreatic ductal adenocarcinoma | 6 | 8 | 2 (4), 4 (4) | Plasma (cell-free DNA) | N/A |  |
|  | Tumor Seeding During Colonoscopy as a Possible Cause for Metachronous Colorectal Cancer | 6 | 5 | pT3N0 (1), pT3N2 (3), pT4N1 (1) | Rectum (3), Sigmoid colon (1), Transverse colon (1) | N/A |  |
|  | Overlooked risk for needle tract seeding following endoscopic ultrasound-guided minimally invasive tissue acquisition ** | 5 | 21 | T1N0M0 (5), T2N0M0 (6), T3N0M0 (5), Unknown (5) | Gastric wall (30), esophageal wall (2), gastroesophageal junction (1) - mainly in submucosa | N/A |  |
|  | Preoperative EUS-guided FNA: effects on peritoneal recurrence and survival in patients with pancreatic cancer | 4 | 131 (27 EUS-FNA, 104 non EUS-FNA) | N/A | Peritoneum | N/A |  |
|  | Needle tract seeding after endoscopic ultrasound-guided tissue acquisition of pancreatic tumors: Nationwide survey in Japan | 4 | 38 PDAC with transgastric EUS-TA | 1(8), 2(23), 3(6), 4(1) | 37 gastric wall, 1 adjacent to gastric wall | N/A |  |
|  | Port site metastasis. An unresolved problem in laparoscopic surgery. A review | 5 | 105 | I (1), II (6), III (13), IV (6) - Dukes A (2), Dukes B (8), Dukes C (14), Dukes D (3), unknown (52) | N/A | N/A |  |
|  | Abdominal wall metastases following laparoscopy | 5 | 18 | N/A | Abdominal wall (port site) | N/A |  |
|  | Does seeding of intraluminal tumour cells cause anastomotic recurrence after oesophageal resection? | 6 | 14 | N/A | N/A | N/A |  |
|  | Exfoliated Tumor Cells in Intraluminal Lavage Samples after Colorectal Endoscopic Submucosal Dissection: A Pilot Study ** | 6 | 2 | N/A | Intestinal lumen | N/A |  |
| Breast cancer (unspecified) | Risk of needle-track seeding after diagnostic image-guided core needle biopsy in breast cancer. | 4 | 15 (11 IGCNB, 4 NLBB) | N/A | Needle tract | N/A | **15** |
| Carcinoma | Port-site metastases in patients undergoing laparoscopy for gastrointestinal malignancy | 4 | 5 (all serosal-involvement) | N/A | Port site | Port-site recurrence may be related to serosal involvement of tumor | **1969** |
|  | Neoplastic Seeding After Radiofrequency Ablation for Hepatocellular Carcinoma | 4 | 33 | Well-differentiated (25), poorly-differentiated (8) | Peritoneum (25), Pleura (7), Abdominal wall (1) | Poor differentiation degree was the risk factor of neoplastic seeding after RFA for HCC. The surrogate markers for poor differentiation degree were larger tumor size and elevated tumor marker levels. |  |
|  | Needle tract implantation of hepatocellular carcinoma and pancreatic carcinoma after ultrasound-guided percutaneous puncture: clinical and pathologic characteristics and the treatment of needle tract implantation | 3 | 6 | N/A | Needle tract | Needle tract implantation develops regardless of the procedure or the pathologic differentiation of the primary tumor |  |
|  | Peritoneal seeding of hepatocellular carcinoma: clinicopathological characteristics of 17 autopsy cases | 4 | 17 | Well (2), Moderate (8), Poor (7) | diaphragm, omentum | Lymph node metastasis of HCC is a risk factors for peritoneal seeding |  |
|  | Risk of needle tract seeding of breast cancer: cytological results derived from core wash material | 4 | 134 | N/A | needle track | The type of carcinoma is one of most important factors for the rate of needle tract seeding; invasive lobular carcinoma was less likely to seed than ductal carcinoma in situ. |  |
|  | Breast cancer neoplastic seeding in the setting of image-guided needle biopsies of the breast | 4 | 8 | T1 (2), T2 (3), T3 (3) |  | Tumor histology appears predictive of risk, but patient size is too small to make a strong conclusion. It appears that invasive ductal carcinoma is a high risk factor in seeding |  |
|  | Treatment and clinical outcome of needle-track seeding from hepatocellular carcinoma | 4 | 8 | N/A | Needle tract (8), skin (4/8) | N/A |  |
|  | Needle tract implantation after sonographically guided percutaneous biopsy of hepatocellular carcinoma: evaluation of doubling time, frequency, and features on CT | 4 | 17 | N/A | Needle tract: subcutaneous tissue (6), intercostal muscles (6), peritoneal cavity (3), continuous or elongated lesion along tract (2) | N/A |  |
|  | Needle track seeding in hepatocellular carcinoma after local ablation by high-dose-rate brachytherapy: a retrospective study of 588 catheter placements | 4 | 9 | N/A | Needle tract: liver (7), peritoneal cavity (2) | N/A |  |
|  | Are malignant cells displaced by large-gauge needle core biopsy of the breast? | 4 | 114 (76 one or two cell clusters, 38 multiple tumor fragments) - 74 automated gun, 13 palpation-guided, 27 vacuum-assisted | T1a (15), T1b (25), T1c (31), T2 (22), T3 (8) | Within needle tract or in stroma immediately adjacent to needle tract | N/A |  |
|  | Subcutaneous metastasis of pancreatic cancer in the site of percutaneous biliary drainage** | 5 | 29 | N/A | Subcutaneous (9), Pleura (2), Skin (1), Liver (1), Abdominal wall (1) | N/A |  |
|  | Comparison of a coaxial versus non-coaxial liver biopsy technique in an oncological setting: diagnostic yield, complications and seeding risk** | 4 | 10 | N/A | PLB tract | N/A |  |
|  | PET/CT detects abdominal wall and port site metastases of colorectal carcinoma | 4 | 12 (8 laparotomy, 4 laparoscope-assisted surgery) | T3N2M1 (1), T3N1M0 (3), T3N2M0 (1), T4N2M0 (1), T3N0M0 (3), T4N0M1 (1), T3N0M1 (1) | Abdominal wall: surgical scar (7), stoma (2), drain (3), laparoscope ports (4) | N/A |  |
|  | Tumour cell displacement after 14G breast biopsy | 4 | 18 | N/A | Needle tract | N/A |  |
|  | Foreign body reaction mimicking local recurrence from polyactide adhesion barrier film after laparoscopic colorectal cancer surgery: A retrospective cohort study | 4 | 8 (6 PLA, 2 non-PLA) | N/A | Peritoneum | N/A |  |
|  | Port site metastases: where are we at the beginning of the 21st century? | 5 | 71 | N/A | Trocar site | N/A |  |
|  | Exfoliated Tumor Cells in Intraluminal Lavage Samples after Colorectal Endoscopic Submucosal Dissection: A Pilot Study** | 6 | 5 | N/A | Intestinal lumen | N/A |  |
|  | Needle tract seeding after radiofrequency ablation of hepatic tumors | 4 | 8 | N/A | Peritoneal cavity (3), subcutaneous (4), port site + peritoneal cavity (1) | N/A |  |
|  | Needle-tract implantation in hepatocellular carcinoma: frequency and CT findings after biopsy with a 19.5-gauge automated biopsy gun | 4 | 7 | N/A | Needle tract | N/A |  |
|  | Breast cancer seeding associated with core needle biopsies: a systematic review | 5 | 927 | N/A | N/A | N/A |  |
|  | Needle tract implantation of hepatocellular carcinoma after fine needle biopsy | 6 | 5 | N/A | Chest wall (3), Subcutaneous (1), Peritoneal/chest wall (1) | N/A |  |
|  | Risk of tumour seeding after percutaneous radiofrequency ablation for hepatocellular carcinoma | 4 | 12 | N/A | Subcutaneous abdominal tissue (3), intercostal muscle (4), parietal peritoneum (4), omentum (1) | N/A |  |
|  | Tumor seeding following laparoscopy: international survey | 4 | 109 | T0 (13), T1a (3), T1b (10), T2 (36), T3 (16), T4 (5), remaining unspecified | Port site | N/A |  |
|  | Epithelial displacement during breast needle core biopsy causes diagnostic difficulties in subsequent surgical excision specimens | 6 | 7 | N/A | breast | N/A |  |
|  | Port site recurrences after laparoscopic surgery. A review | 6 | 164 | T1 (9), T2 (33), T3 (3), T4 (1) | port site | N/A |  |
|  | The use of positive core wash cytology to estimate potential risk of needle tract seeding of breast cancer: directional vacuum-assisted biopsy versus automated core needle biopsy | 4 | 102 (CNB) vs 35 (vacuum-assisted biopsy) | N/A | Needle tract | N/A |  |
|  | Recurrent gallbladder carcinoma along laparoscopic cholecystectomy port tracks: CT demonstration | 6 | 6 | N/A | Port site | N/A |  |
|  | Peritoneal seeding in intraductal papillary mucinous neoplasm of the pancreas patients who underwent endoscopic ultrasound-guided fine-needle aspiration: the PIPE Study | 4 | 7 | T2 (2), T3 (4) | Peritoneum | N/A |  |
|  | The rate of port-site metastases after 2251 laparoscopic procedures in women with underlying malignant disease | 4 | 20 | IB (2), IIIC (16), IV (2) | Port site | N/A |  |
|  | Wound recurrence following conventional treatment of colorectal cancer. A rare but perhaps underestimated problem | 4 | 26 | B2 (5), C1 (13), C2 (8) | Skin, abdominal wall, perineum | Incisional recurrence is likely associated with diffuse intra-abdominal disease |  |
|  | Trocar site recurrence in laparoscopic surgery for colorectal cancer: myth or real concern? | 5 | 17 | N/A | Port site | N/A |  |
|  | Overlooked risk for needle tract seeding following endoscopic ultrasound-guided minimally invasive tissue acquisition ** | 5 | 12 | T2N2M0 (1)  T3N0M0 (2),  T3N0M1 (1), T4N1M0 (1),  Unknown (7) | Gastric wall (30), esophageal wall (2), gastroesophageal junction (1) - mainly in submucosa | N/A |  |
|  | Trans-peritoneal fine needle aspiration biopsy of hilar cholangiocarcinoma is associated with disease dissemination | 4 | 5 | N/A | Peritoneum | N/A |  |
| Colorectal cancer  (unspecified) | Seeding after ultrasound-guided percutaneous biopsy of liver metastases in patients with colorectal or breast cancer | 4 | 17 | N/A | Abdominal wall | N/A | **282** |
|  | Port-site metastasis after laparoscopic surgery for gastrointestinal cancer | 1 | 58 | N/A | Port site | N/A |  |
|  | Disseminated single tumor cells as detected by real-time quantitative polymerase chain reaction represent a prognostic factor in patients undergoing surgery for colorectal cancer | 3 | **16** (5 disease recurrence, 11 +qPCR dissemination) | 1 (1), 2 (1), 3 (9) | Peritoneal lavage fluid (7), peripheral and mesenteric venous blood (4) | N/A |  |
|  | Biopsy of resectable colorectal liver metastases causes tumour dissemination and adversely affects survival after liver resection | 4 | 17 | 4 | Needle tract | N/A |  |
|  | Colonic perforation either during or after stent insertion as a bridge to surgery for malignant colorectal obstruction increases the risk of peritoneal seeding | 4 | 6 (5 stent, 1 surgery-alone) | IIB (2), IIIB/IVA (3) - stent | Intra-abdominal | N/A |  |
|  | Extent of intraluminal exfoliated malignant cells during surgery for colon cancer: Differences in cell abundance ratio between laparoscopic and open surgery | 4 | 27 total: 8 open (2 proximal, 6 distal), 21 laparoscopic (14 proximal, 7 distal) [can be overlap in numbers] | Overall staging provided, not for seeding cases alone | Intra-luminal | N/A |  |
|  | Increased detection rate and potential prognostic impact of disseminated tumor cells in patients undergoing endorectal ultrasound for rectal cancer | 4 | 11 | 1 (3), 2 (1), 3 (5), N/A (2) | Peripheral venous blood | N/A |  |
|  | Risk of dissemination with biopsy of colorectal liver metastases | 4 | 7 | N/A | Peritoneum, small bowel, subcutaneous at site of biopsy | N/A |  |
|  | Risk factors contributing to early occurrence of port site metastases of laparoscopic surgery for malignancy** | 5 | 49 | N/A | Port site | Most port site metastases (PSM) were adenocarcinomas, advanced stage, and with diffuse peritoneal carcinomatosis. |  |
|  | Tumor recurrence in the abdominal wall scar tissue after large-bowel cancer surgery. | 6 | 11 | Duke’s B (8), Duke’s C1 (3) | Abdominal wall scar tissue | N/A |  |
|  | The theories and realities of port-site metastases: a critical appraisal. | 5 | 28 | N/A | Port site | N/A |  |
|  | Port site recurrences after laparoscopic and thoracoscopic procedures in malignancy** | 5 | 35 | 3 A, 9 B, 12 C, 2 D (Duke's staging system) | Port site | N/A |  |
| Gallbladder cancer  (unspecified) | Risk factors contributing to early occurrence of port site metastases of laparoscopic surgery for malignancy** | 5 | 12 | N/A | Port site | Most port site metastases (PSM) were adenocarcinomas, advanced stage, and with diffuse peritoneal carcinomatosis. | **24** |
|  | Port site recurrences after laparoscopic and thoracoscopic procedures in malignancy** | 5 | 12 | Tis (1), T2 (3), T3 (8), T4 (1) | Port site | N/A |  |
| Gastric cancer  (unspecified) | Predicting factors of unexpected peritoneal seeding in locally advanced gastric cancer: indications for staging laparoscopy | 4 | 72 (staging laparoscopy: 60, direct laparotomy: 12) | cT stage: T2 (7), T3 (54), T4 (11). cN stage: N0 (6), N1 (29), N2 (37) | Peritoneum | Borrmann type 3 (OR: 4.475) or type 4 (OR: 8.243) cancer, tumor invasion of T3 (OR: 2.794) or T4 (OR: 6.841) and tumor size (4 cm ≤ tumor size < 8 cm; OR: 3.723 and 8 cm ≤ tumor size; OR: 6.971) were predictive factors for overall peritoneal seeding. | **113** |
|  | Influence of laparoscopic gastrectomy on the detection rate of free gastric cancer cells in the peritoneal cavity | 4 | 31 (recurrent disease): 15 laparoscopic, 16 open | 1 (19), 2 (22), 3 (16), 4 (11) | Peritoneal washings (+ cytology); peritoneal cavity | N/A |  |
|  | Surgical Treatment of Port-Site Metastases After Laparoscopic Radical Resection of Gastrointestinal Tumors | 4 | 8 | T3 (4), T4 (4) | port site | N/A |  |
|  | Risk factors contributing to early occurrence of port site metastases of laparoscopic surgery for malignancy** | 5 | 1 | N/A | Port site | Most port site metastases (PSM) were adenocarcinomas, advanced stage, and with diffuse peritoneal carcinomatosis. |  |
|  | Comparison of a coaxial versus non-coaxial liver biopsy technique in an oncological setting: diagnostic yield, complications and seeding risk ** | 4 | 1 | N/A | PLB tract | N/A |  |
| Gastrointestinal cancer (unspecified) | Implantation metastases from gastrointestinal cancer after percutaneous puncture or biliary drainage | 6 | 8 | N/A | Needle track (2), subcutaneous (2), subcutaneous and needle track (4) | N/A | **8** |
| Hepatic cancer (unspecified) | Risk factors contributing to early occurrence of port site metastases of laparoscopic surgery for malignancy** | 5 | 2 | N/A | Port site | Most port site metastases (PSM) were adenocarcinomas, advanced stage, and with diffuse peritoneal carcinomatosis. | **2** |
| Liposarcoma | Needle tract seeding following core biopsies in retroperitoneal sarcoma | 4 | 5 | N/A | needle tract | N/A | **5** |
| Melanoma | Comparison of a coaxial versus non-coaxial liver biopsy technique in an oncological setting: diagnostic yield, complications and seeding risk ** | 4 | 2 | N/A | PLB tract | N/A | **2** |
| Osteosarcoma | Tumor cell seeding in the biopsy tract and its clinical significance in osteosarcomas | 4 | 11 | N/A | needle tract | Osteosarcoma appears to be a high risk tumor for seeding, with 20% of patients who underwent biopsy showing seeding | **11** |
| Pancreatic cancer  (unspecified) | Risk of peritoneal carcinomatosis by endoscopic ultrasound-guided fine needle aspiration for pancreatic cancer | 4 | 34 (24 ERCP, 10 ERCP + EUS-FNA) | N/A | Peritoneum | N/A | **35** |
|  | Risk factors contributing to early occurrence of port site metastases of laparoscopic surgery for malignancy** | 5 | 1 | N/A | Port site | Most port site metastases (PSM) were adenocarcinomas, advanced stage, and with diffuse peritoneal carcinomatosis. |  |

| **Gynecologic oncology** | | | | | | | |
| --- | --- | --- | --- | --- | --- | --- | --- |
| **Tumor cytology** | **Title** | **LOE** | **Seeding (n=)** | **Staging (n=)** | **Location of seeded tumor** | **Cytology-related risk?** | **Total (n=)** |
| Adenocarcinoma | Port-site metastasis after laparoscopic surgical staging of endometrial cancer: a systematic review of the published and unpublished data | 5 | 12 | IA (1), IB (1), IC (2)  IIB (2)  IIIA (2), IIIC (3)  IV (1) | Port site | Port site metastases of endometrial cancer rarely occur but are likely an expression of aggressive disease. | **153** |
|  | Port-site metastasis after laparoscopic surgery for gynecologic cancer. A report of six cases | 6 | 6 | III (4)  IV (1)  N/A (1) | Port site | N/A |  |
|  | Low incidence of port-site metastases after laparoscopic staging of uterine cancer** | 4 | 1 | IVA (1) | Port site | All patients with port site metastases had concomitant metastases. |  |
|  | The implantation of cervical neoplasia at postpartum episiotomy scar: the clinical evidence ** | 5 | 5 | IB (5) | Postpartum episiotomy scar | N/A |  |
|  | Port site metastases after robot-assisted surgery: a systematic review ** | 5 | 2 | IB1 (1)  III grade 1 (1) | Port site | N/A |  |
|  | Iatrogenic transtubal spill of endometrial cancer: risk or myth | 5 | 127 (47 hysteroscopy, 36 D&C, 16 SIS, 15 laparoscopy, 12 H/S + D&C, 1 SIS + D&C) | N/A | Peritoneal lavage fluid | N/A |  |
| Carcinoma | The implantation of cervical neoplasia at postpartum episiotomy scar: the clinical evidence | 5 | 13 | IB (1), IA (1), IB (7), 1B2 (1), IIIA (2), IIIB (1) | Postpartum episiotomy scar | N/A | **107** |
|  | Laparoscopic port-site metastases in patients with gynecological malignancies | 5 | 58 (ovarian carcinoma) | N/A | Port site | Port-site metastases can occur in early or late stage disease. In this study, 83% of patients had advanced (III/IV) disease. |  |
|  | Does hysteroscopy facilitate tumor cell dissemination? Incidence of peritoneal cytology from patients with early stage endometrial carcinoma following dilatation and curettage (D & C) versus hysteroscopy and D & C | 4 | 10 (endometrial carcinoma) | I (2)  II (6)  III (1)  N/A (1) | Peritoneal washings | N/A |  |
|  | Port site metastases after laparoscopic lymph node staging of cervical carcinoma | 6 | 6 (cervical carcinoma) | N/A | Port site | N/A |  |
|  | Low incidence of port-site metastases after laparoscopic staging of uterine cancer | 4 | 4 | IB (1), IC (1) IIB (2) | Port site | All patients with port site metastases had concomitant metastases. |  |
|  | Subcutaneous tumor implantation after laparoscopic procedures in women with malignant disease | 4 | 13 (6 papillary serous, 4 endometrioid, 2 ductal and/or lobular, 1 squamous) | Recurrent IB (1)  3A (1)  3C (9)  IV (2) | Subcutaneous | Subcutaneous implantation appears to occur in patients with known metastatic disease |  |
|  | The implantation of cervical neoplasia at postpartum episiotomy scar: the clinical evidence ** | 5 | 12 | IA (1)  IB (7)  1B2 (1)  IIIA (2)  IIIB (1) | Postpartum episiotomy scar | N/A |  |
|  | Port site metastases after robot-assisted surgery: a systematic review ** | 5 | 2 | IB (1), T3bN0M0 (1) | Port site | N/A |  |
| Leiomyosarcoma | Impact of morcellation on survival outcomes of patients with unexpected uterine leiomyosarcoma: A systematic review and meta-analysis. | 5 | 39 (24 morcellated, 15 non-morcellated) | N/A | Intra-abdominal | N/A | **39** |
| Ovarian cancer, endometrial cancer, cervical cancer (unspecified) | Risk factors contributing to early occurrence of port site metastases of laparoscopic surgery for malignancy** | 5 | 20 (17 ovarian, 2 cervical, 1 endometrial) | N/A | Port site | Most port site metastases (PSM) were adenocarcinomas, advanced stage, and with diffuse peritoneal carcinomatosis. Ovarian cancer was a risk factor. | **27** |
|  | Port site recurrences after laparoscopic and thoracoscopic procedures in malignancy** | 5 | 7 | T2a (1), T3c (4), low malignant potential (4) | Port site | N/A |  |
| Uterine mesenchymal neoplasms  (unspecified) | Peritoneal dissemination complicating morcellation of uterine mesenchymal neoplasms. | 4 | 12 | N/A | Peritoneum | Of endometrial stromal sarcoma, cellular or atypical leiomyomas, STUMPs, and leiomyosarcoma, only disseminated leiomyosarcoma was associated with mortality | **12** |

| **Ophthalmology** | | | | | | | |
| --- | --- | --- | --- | --- | --- | --- | --- |
| **Tumor cytology** | **Title** | **LOE** | **Seeding (n=)** | **Staging (n=)** | **Location of seeded tumor** | **Cytology-related risk?** | **Total (n=)** |
| Melanoma | Quantitation of tumor seeding from fine needle aspiration of ocular melanomas | 6 | 8 | N/A | Needle track: episclera (5), vitreous/aqueous humor (2), scleral blood vessel (1) | N/A | **8** |

| **Orthopedic Surgery** | | | | | | | |
| --- | --- | --- | --- | --- | --- | --- | --- |
| **Tumor cytology** | **Title** | **LOE** | **Seeding (n=)** | **Staging (n=)** | **Location of seeded tumor** | **Cytology-related risk?** | **Total (n=)** |
| Sarcoma (bone/soft tissue) | Are Biopsy Tracts a Concern for Seeding and Local Recurrence in Sarcomas? | 4 | 21 (20 open biopsy, 1 percutaneous core needle biopsy) | N/A | Biopsy tract | N/A | **29** |
|  | Needle tract seeding after percutaneous biopsy of sarcoma: Risk/benefit considerations | 5 | 8 (malignant cells in needle tract; no clinically significant disease) | N/A | Biopsy tract | N/A |  |

| **Otolaryngology – Head and Neck Surgery** | | | | | | | |
| --- | --- | --- | --- | --- | --- | --- | --- |
| **Tumor cytology** | **Title** | **LOE** | **Seeding (n=)** | **Staging (n=)** | **Location of seeded tumor** | **Cytology-related risk?** | **Total (n=)** |
| Adenocarcinoma | Recurrence of Ventral Skull Base Lesions Attributed to Tumor Seeding: A Systematic Review ** | 5 | 3 | N/A | Surgical access pathway | N/A | **8** |
|  | Metastatic spread to a percutaneous gastrostomy site from head and neck cancer: case report and literature review** | 6 | 1 | N/A | PEG tube site | N/A |  |
|  | Metastasis of head and neck carcinoma to the site of percutaneous endoscopic gastrostomy: case report and literature review** | 6 | 1 | N/A | PEG exit site | N/A |  |
|  | Risk factors and risk reduction of malignant seeding of the percutaneous endoscopic gastrostomy track from pharyngoesophageal malignancy: a review of all 44 known reported cases** | 5 | 1 | I/II | Abdominal wall / PEG exit site (63%), gastric wall / PEG entrance site (7%), Both walls (30%) | Strong risk factors for stomal metastases: squamous cell histology, less well-differentiated cancer, large size, advanced stage. |  |
|  | Percutaneous endoscopic gastrostomy site metastasis | 6 | 2 | N/A | PEG exit site | N/A |  |
| Carcinoma | Recurrence of Ventral Skull Base Lesions Attributed to Tumor Seeding: A Systematic Review ** | 5 | 2 | N/A | Surgical access pathway | N/A | **452** |
|  | Needle tract implantation of papillary thyroid carcinoma after fine-needle aspiration biopsy | 4 | 10 | 6 poorly differentiated, 3 well differentiated, 1 unknown | Needle tract: subcutaneous tissue, intra-strap muscle | High growth activity in the metastatic lesions may be a risk factor of FNAB |  |
|  | A systematic review of cases reporting needle tract seeding following thyroid fine needle biopsy | 5 | 9 | N/A | Cutaneous (7), sternocleidomastoid (2) | N/A |  |
|  | Unexpected High Rate of Malignant Seeding by Percutaneous Endoscopic Gastrostomy (PEG) Implantation: Final Results of a Prospective Study? | 4 | 9 | N/A | Abdominal wall (PEG outlet) | Increased risk with higher tumor stages and squamous cell cancers, for which direct PEG tube placement should be favored |  |
|  | Percutaneous endoscopic gastrostomy site metastasis from head and neck squamous cell carcinoma: case series and literature review | 5 | 42 | I/II (2)  III/IV (35)  N/A (5) | Abdominal wall | N/A |  |
|  | Tumor implantation following percutaneous endoscopic gastrostomy insertion for head and neck and oesophageal cancer: Review of the literature ** | 5 | 85 | N/A | PEG tube exit site; stomach | N/A |  |
|  | Metastasis of untreated head and neck cancer to percutaneous gastrostomy tube exit sites | 5 | 46 | II (1)  III (7)  IV (38) | PEG tube exit site | N/A |  |
|  | Risk factors and risk reduction of malignant seeding of the percutaneous endoscopic gastrostomy track from pharyngoesophageal malignancy: a review of all 44 known reported cases** | 5 | 43 | III/IV | Abdominal wall / PEG exit site (63%), gastric wall / PEG entrance site (7%), Both walls (30%) | Strong risk factors for stomal metastases: squamous cell histology, less well-differentiated cancer, large size, advanced stage. |  |
|  | Recurrence of Ventral Skull Base Lesions Attributed to Tumor Seeding: A Systematic Review ** | 5 | 2 | N/A | Surgical access pathway | N/A |  |
|  | Metastatic spread to a percutaneous gastrostomy site from head and neck cancer: case report and literature review** | 6 | 29 | II (1)  III (4)  IV (25) | PEG tube site | N/A |  |
|  | Metastasis of head and neck carcinoma to the site of percutaneous endoscopic gastrostomy: case report and literature review** | 6 | 19 | T1 (1), T2 (2), T3 (1), T4 (10) | PEG exit site | N/A |  |
|  | Risk of tumor implantation in percutaneous endoscopic gastrostomy in the upper aerodigestive tumors | 5 | 50 | N/A | PEG exit site | Older patients and higher tumor stages show a greater incidence of recurrence after PEG tube placement in patients with head and neck and esophageal malignancy. |  |
|  | Percutaneous endoscopic gastrostomy site metastasis ** | 6 | 45 | N/A | PEG exit site | N/A |  |
|  | Malignant Seeding Following Percutaneous Endoscopic Gastrostomy Placement in Head and Neck Cancer: Review of Literature | 6 | 61 | N/A (stage IIIA - IV B had increased incidence) | PEG exit site | N/A |  |
| Carcinosarcoma | Tumor implantation following percutaneous endoscopic gastrostomy insertion for head and neck and oesophageal cancer: Review of the literature ** | 5 | 4 | N/A | PEG tube exit site; stomach | N/A | **4** |
| Chordoma | Surgical pathway seeding of clivo-cervical chordomas | 5 | 14 | N/A | Maxilla (3), nasal cavity or septum (6) palate (2), parotid (1), maxillary sinus, neck (2), abdomen (2), petrous apex/retroauricular area (1), cervical spine (2) | N/A | **71** |
|  | Tumour seeding in the surgical pathway after resection of skull base chordoma | 5 | 34 | N/A | Surgical pathway: neck (5), soft/hard palate (3), sinuses (3), nasal cavity/incision/septum (11), other head and neck (12) | N/A |  |
|  | Recurrence of Ventral Skull Base Lesions Attributed to Tumor Seeding: A Systematic Review ** | 5 | 23 | N/A | Surgical access pathway | Chordoma appears to be especially high risk of iatrogenic seeding. |  |
| Craniopharyngioma | Recurrence of Ventral Skull Base Lesions Attributed to Tumor Seeding: A Systematic Review ** | 5 | 36 | N/A | Surgical access pathway | Craniopharyngioma appears to be especially high risk of iatrogenic seeding. | **36** |
| Head and neck cancer (unspecified) | Incidence of abdominal wall metastases following percutaneous endoscopic gastrostomy placement in patients with head and neck cancer | 4 | 5 | IV (5) | Abdominal wall | N/A | **36** |
|  | Risk of Esophageal Cancer Following Percutaneous Endoscopic Gastrostomy in Head and Neck Cancer Patients: A Nationwide Population-Based Cohort Study in Taiwan | 4 | 31 (16 PEG, 15 control) | N/A | Esophagus | N/A |  |
| Metastatic melanoma, pleomorphic adenoma, adenocarcinoma, squamous cell carcinoma | Tumour seeding after fine-needle aspiration and core biopsy of the head and neck--a systematic review | 5 | 7 | N/A | Needle tract | N/A | **7** |

| **Thoracic Surgery** | | | | | | | |
| --- | --- | --- | --- | --- | --- | --- | --- |
| **Tumor cytology** | **Title** | **LOE** | **Seeding (n=)** | **Staging (n=)** | **Location of seeded tumor** | **Cytology-related risk?** | **Total (n=)** |
| Adenocarcinoma | Risk of pleural recurrence after needle biopsy in patients with resected early stage lung cancer | 4 | 10 | P0 (5), P1 (3), P2 (2) | Pleura (9), needle tract (1) | N/A | **16** |
|  | Dissemination of malignant tumors after video assisted thoracic surgery: A report of twenty-one cases.** | 6 | 6 | N/A | Incision, staple line, pleura | N/A |  |
| Lung cancer (unspecified) | Preoperative percutaneous needle lung biopsy techniques and ipsilateral pleural recurrence in stage I lung cancer | 4 | 40 | IA (9), IB (31) | Pleura | N/A | **90** |
|  | Intraoperative fine-needle aspiration biopsy (FNA) for lung cancer: diagnostic value and risk of pleural dissemination | 4 | 21 | N/A | Pleura | N/A |  |
|  | CT-guided needle biopsy of lung lesions: a survey of severe complication based on 9783 biopsies in Japan | 4 | 6 | N/A | Port site | N/A |  |
|  | Port site recurrences after laparoscopic and thoracoscopic procedures in malignancy** | 5 | 23 | N/A | Port site | N/A |  |
| Metastases to the lung | Dissemination of malignant tumors after video assisted thoracic surgery: A report of twenty-one cases.** | 6 | 6 | N/A | Incision, staple line, pleura | N/A | **6** |
| Non-small cell lung cancer (NSCLC) | Risk of pleural recurrence after percutaneous transthoracic needle biopsy in stage I non-small-cell lung cancer | 4 | 68 PTNB | I (68) | Pleura | N/A | **294** |
|  | Management for chest wall implantation of non-small cell lung cancer after fine-needle aspiration biopsy | 4 | 9 | Ib (7) IIIa (1) IV (1) | Chest wall | N/A |  |
|  | Pleural recurrence and long-term survival after thoracotomy and thoracoscopic lobectomy | 4 | 26 (14 VATS,  12 thoracotomy) | N/A | Pleura | N/A |  |
|  | Is needle biopsy a risk factor of pleural recurrence after surgery for non-small cell lung cancer? | 4 | 191 | N/A | Pleura | Pathological lymph node factor is an independent risk factor for pleural recurrence |  |
| Pleural mesothelioma | Pleural mesothelioma: sensitivity and incidence of needle track seeding after image-guided biopsy versus surgical biopsy | 4 | 16 | N/A | Subcutaneous (scar site) | N/A | **67** |
|  | Prophylactic irradiation of intervention sites in malignant pleural mesothelioma | 4 | 46 (6 PIT, 40 non-PIT) | N/A | Subcutaneous | N/A |  |
|  | Dissemination of malignant tumors after video assisted thoracic surgery: A report of twenty-one cases.** | 6 | 5 | N/A | Incision, staple line, pleura | N/A |  |
| Small cell lung cancer | Dissemination of malignant tumors after video assisted thoracic surgery: A report of twenty-one cases.** | 6 | 1 | N/A | Incision, staple line, pleura | N/A | **1** |
| Squamous cell carcinoma & adenocarcinoma | Incidence of pleural recurrence after computed tomography-guided needle biopsy in stage I lung cancer | 4 | 8 | I (8) | Pleura | N/A | **128** |
|  | Risk of pleural recurrence after computed tomographic-guided percutaneous needle biopsy in stage I lung cancer patients | 4 | 8 | I (8) | Pleura | Stage 1B cases with subpleural lesions may have increased risk of pleural implantation following CTGNB |  |
|  | Fine-needle aspiration cytologic technique for lung cancer has a high potential of malignant cell spread through the tract | 4 | 20 | N/A | Needle tract | N/A |  |
|  | Dissemination of malignant tumors after video assisted thoracic surgery: A report of twenty-one cases.** | 6 | 4 | N/A | Incision, staple line, pleura | N/A |  |
|  | Needle-track metastasis after transthoracic needle biopsy | 5 | 27 | N/A | Needle tract | N/A |  |
|  | Procedure-related tumour seeding in lung cancer with malignant pleural effusion: Radiological features and outcomes | 4 | 37 | N/A | Pleura | N/A |  |
|  | Video-assisted thoracic surgery (VATS) for cancer. Risk of parietal seeding and of early local recurrence | 5 | 24 | N/A | Surgical incision (16), parietal pleura (2), pleural and incisional (1), pulmonary staple line (2), pleural and staple line (1), incisional and staple line (1), pleural with invasion of lung and chest wall (1) | N/A |  |

| **Urology** | | | | | | | |
| --- | --- | --- | --- | --- | --- | --- | --- |
| **Tumor cytology** | **Title** | **LOE** | **Seeding (n=)** | **Staging (n=)** | **Location of seeded tumor** | **Cytology-related risk?** | **Total (n=)** |
| Adenocarcinoma | Needle biopsy associated tumor tracking of adenocarcinoma of the prostate | 4 | 7 | Clinical stage B (7):  Intermediate (6)  High (1) | Needle tract (3)  Local soft tissue (4) | N/A | **58** |
|  | Incidence of needle-tract seeding following prostate biopsy for suspected cancer: a review of the literature | 5 | 42 | Low grade (2)  Intermediate grade (7)  High grade (12) | Perineum | N/A |  |
|  | Seeding and perineal implantation of prostatic cancer in the track of the biopsy needle: three case reports and a review of the literature ** | 5 | 9 | N/A | Perineal subcutaneous tissue | N/A |  |
| Carcinoma | Seeding and perineal implantation of prostatic cancer in the track of the biopsy needle: three case reports and a review of the literature ** | 5 | 5 | N/A | Perineal subcutaneous tissue | N/A | **2133** |
|  | Port site metastasis and tumor seeding in oncologic laparoscopic urology ** | 5 | 22 | T1bN0M0 (1)  T1N0M0G3 (1)  T1N0G2 (2)  T2N0M0 (2)  T3N0G4 (1)  T2G3 (2)  pT3N0M0 (1)  N/A (1)  T1G2 (1)  T1G3 (4)  T3bN0M0G3 (1)  T3G2 (2)  T3G3 (2)  G3 (1) | Port site | N/A |  |
|  | Tumour Seeding in the Tract of Percutaneous Renal Tumour Biopsy: A Report on Seven Cases from a UK Tertiary Referral Centre | 6 | 7 (6 papillary RCC, 1 clear cell RCC) | pT3a (7) | PEG tube exit site; stomach | N/A |  |
|  | Needle track seeding in renal mass biopsies | 5 | 16 (14 papillary RCC, 1 clear cell RCC, 1 N/A) | N/A | Needle tract | Needle tract seeding may be associated with tumor upstaging and papillary RCC. |  |
|  | Port site metastasis after surgery for renal cell carcinoma: harbinger of future metastasis | 5 | 16 | Fuhrman grade 3 or higher (16) | Port site | High tumor grade appears to contribute to tract seeding |  |
|  | Needle tract seeding in renal tumor biopsies: experience from a single institution | 4 | 6 | pT1a (6) | Needle tract | N/A |  |
|  | Tumor seeding in urological laparoscopy: an international survey | 4 | 9 | pT2/G3 (1), T1 (2): G2 (1), G3 (1)  T2 (1): G3 (1), N/A (2) | Peritoneum | Tumors with high grade histology are at a greater risk for dissemination. |  |
|  | Port site metastases | 5 | 28 | G2 (7)  G3 (13)  G4 (2) | Port site | Tumor aggressiveness appears to be a significant factor. |  |
|  | Diagnostic Ureteroscopy Prior to Radical Nephroureterectomy for Upper Tract Urothelial Carcinoma Increased the Risk of Intravesical Recurrence | 5 | 1403 | N/A | Bladder (intravesicular) | N/A |  |
|  | Diagnostic Ureterorenoscopy Is Associated with Increased Intravesical Recurrence following Radical Nephroureterectomy in Upper Tract Urothelial Carcinoma | 4 | 268 | T1 (84)  T2 (48)  T3/4 (91)  Ta (59) | Bladder (intravesicular) | History of bladder tumour, extravesical excision of distal ureter, multifocal tumour, and URS confer higher risk. |  |
|  | The Impact of Upper Tract Urothelial Carcinoma Diagnostic Modality on Intravesical Recurrence after Radical Nephroureterectomy: A Single Institution Series and Updated Meta-Analysis | 4 | 241 | cTa (24)  cT1 (6)  cT2+ (10)  Others N/A | Bladder (intravesicular) | N/A |  |
|  | Risk Factors for Intravesical Recurrence after Minimally Invasive Nephroureterectomy for Upper Tract Urothelial Cancer (ROBUUST Collaboration) | 4 | 112 | Ta (29)  Tis (3)  T1 (26)  T2 (21)  T3 (30)  T4 (1) | Bladder (intravesicular) | N/A |  |
| Germ cell tumor | Port site metastasis and tumor seeding in oncologic laparoscopic urology ** | 5 | 2 | T3N0M0 (1)  IIC (1) | Port site | N/A | **2** |
| Leiomyosarcoma | Seeding and perineal implantation of prostatic cancer in the track of the biopsy needle: three case reports and a review of the literature ** | 5 | 1 | N/A | Perineal subcutaneous tissue | N/A | **1** |
| Prostate cancer (unspecified) | Risk factors for perineal seeding of prostate cancer after needle biopsy | 4 | 5 | “Poor” (3)  “Moderately” (1)  “Well” (1) | Perineum | N/A | **6** |
|  | Port site metastasis and tumor seeding in oncologic laparoscopic urology ** | 5 | 1 | T3N1 (1) | Port site | N/A |  |

| **Dermatology** | | | | | | | |
| --- | --- | --- | --- | --- | --- | --- | --- |
| **Tumor cytology** | **Title** | **LOE** | **Seeding (n=)** | **Staging (n=)** | **Location of seeded tumor** | **Cytology-related risk?** | **Total (n=)** |
| Melanoma | Does needle biopsy cause an increased risk of extracapsular extension in the diagnosis of metastatic lymph node in melanoma? | 4 | 28 (needle biopsy), 14 (surgical adenectomy) | N/A | Extracapsular extension of lymph node | N/A | **28** |
