# Supplementary material for: Tumor seeding across specialties: a systematic review
Source: Front Oncol. 2024 Nov 13;14:1464767. doi: 10.3389/fonc.2024.1464767 (PMC11598697; doi:10.3389/fonc.2024.1464767)
Supplement: Supplementary file 3 [file DataSheet3.docx]

| **Thoracic surgery** | | | | | | |
| --- | --- | --- | --- | --- | --- | --- |
| **Instrumentation** | **Title** | **Year** | **LOE** | **Supports biological existence of seeding?** | **Supports clinical importance of seeding?** | **Conclusions** |
| Percutaneous needle biopsy | Needle-track metastasis after transthoracic needle biopsy | 1998 | 5 | Y | N | The incidence of NTM after TNB is approximately 0.012%. This small risk is random and unavoidable. |
|  | Fine-needle aspiration cytologic technique for lung cancer has a high potential of malignant cell spread through the tract | 2000 | 4 | Y | Y | This group showed a 60% frequency of seeding after FNAC of lung cancer, which suggests that FNAC is highly likely to induce seeding in the pleural cavity. |
|  | Management for chest wall implantation of non-small cell lung cancer after fine-needle aspiration biopsy | 2003 | 4 | Y | N | The incidence of chest-wall implantation metastasis after fine-needle aspiration biopsy is extremely rare. With successful resection, the prognosis for the patient seems to depend on the primary cancer. A radical and wide resection in conjunction with irradiation may provide long-term survival in patients with an initial early stage cancer. |
|  | Risk of pleural recurrence after needle biopsy in patients with resected early stage lung cancer | 2005 | 4 | Y | Y | The rate of pleural recurrence for the cases diagnosed by PNB was significantly higher than for the cases diagnosed by bronchoscopy (9.1% versus 1.0%, p 0.0028) or other diagnostic methods (8.6% vs. 0.9%, p=0.0009). |
|  | CT-guided needle biopsy of lung lesions: a survey of severe complication based on 9783 biopsies in Japan | 2006 | 4 | Y | Y | This paper suggests that risk of seeding is correlated with size of needle. |
|  | Pleural mesothelioma: sensitivity and incidence of needle track seeding after image-guided biopsy versus surgical biopsy | 2006 | 4 | Y | N | Image-guided core-needle biopsy has a much lower propensity to cause needle tract seeding (4%) compared with surgical biopsy (22%) in pleural mesothelioma |
|  | Risk of pleural recurrence after computed tomographic-guided percutaneous needle biopsy in stage I lung cancer patients | 2011 | 4 | Y | Y | The CTGNB procedure might increase the risk of pleural implantation in stage I lung cancer patients, especially p stage IB cases with subpleural lesions. |
|  | Incidence of pleural recurrence after computed tomography-guided needle biopsy in stage I lung cancer | 2012 | 4 | Equivocal | N | Pleural recurrence was not significantly increased after CTNB in p-stage I lung cancer patients in this study. |
|  | Intraoperative fine-needle aspiration biopsy (FNA) for lung cancer: diagnostic value and risk of pleural dissemination | 2015 | 4 | Y | N | Rate of recurrence among patients that underwent biopsy (11/147) was not significantly different from patients who did not undergo biopsy (10/177) (p=0.5046) |
|  | Risk of pleural recurrence after percutaneous transthoracic needle biopsy in stage I non-small-cell lung cancer | 2019 | 4 | N | N | PTNB did not significantly increase the risk of pleural recurrence in stage I NSCLC; visceral pleural invasion was responsible for pleural recurrence. |
|  | Is needle biopsy a risk factor of pleural recurrence after surgery for non-small cell lung cancer? | 2020 | 4 | Y | Y | At this institution, FNA was not identified as a risk factor in recurrence of lung cancer. Preoperative computed tomography-guided needle biopsy  however showed significant risk of seeding. |
|  | Preoperative percutaneous needle lung biopsy techniques and ipsilateral pleural recurrence in stage I lung cancer | 2022 | 4 | Y | N | No particular PTNB techniques (needle gauge, biopsy, pleural passage) were associated with reduced pleural seeding after PTNB in stage I lung cancer. |
| Pleural procedures (intercostal tube drainage, thoracentesis, pleural biopsy, pleural thoracoscopy/thoracotomy, chest tube placement) | Port site recurrences after laparoscopic and thoracoscopic procedures in malignancy | 1996 | 5 | Y | Equivocal | Until valid prospective data on PSR frequency are available, laparoscopic or thoracoscopic resection of malignancy off-protocol should be undertaken with circumspection. |
|  | Prophylactic irradiation of intervention sites in malignant pleural mesothelioma | 2011 | 4 | Y | Y | This study suggests that prophylactic irradiation of tracts in mesothelioma following thoracoscopy, thoracotomy, and chest tube placement reduces procedure tract metastasis. |
|  | Procedure-related tumour seeding in lung cancer with malignant pleural effusion: Radiological features and outcomes | 2018 | 4 | Y | Y | Pleural procedure-related tumour seeding in lung cancer with malignant pleural effusion is common. There was a significantly increased risk of death with tumour seeding. Intercostal drainage was an independent predictor of tumour seeding. |
| Video-assisted thoracoscopic surgery (VATS) | Dissemination of malignant tumors after video assisted thoracic surgery: A report of twenty-one cases. | 1996 | 6 | Y | Equivocal | The intractable problem may be tumor disruption, leading to implantation along the margin of resection or on the pleural surface. However, pleural or chest wall implants are rare, suggesting that microscopic deposits may be only marginally viable. Current thoracoscopic techniques may lead to the deposition of larger, more viable clumps of tissue. |
|  | Video-assisted thoracic surgery (VATS) for cancer. Risk of parietal seeding and of early local recurrence | 1996 | 5 | Y | Equivocal | Parietal seeding and early local recurrence have been anecdotally (24 cases) reported after VATS for cancer. Not all cases were undoubtedly related to the thoracoscopic approach itself – strict adherence to principles of carcinologic surgery should minimize these risks. |
|  | Pleural recurrence and long-term survival after thoracotomy and thoracoscopic lobectomy | 2013 | 4 | Y | N | Compared with thoracotomy, VATS lobectomy does not seem to increase the risk of procedure-related pleural dissemination in patients with NSCLC with visceral pleural invasion. |

| **Otolaryngology – Head and Neck Surgery** | | | | | | |
| --- | --- | --- | --- | --- | --- | --- |
| **Instrumentation** | **Title** | **Year** | **LOE** | **Supports biological existence of seeding?** | **Supports clinical importance of seeding?** | **Conclusions** |
| PEG tube placement (pull vs. push technique) | Metastasis of head and neck carcinoma to the site of percutaneous endoscopic gastrostomy: case report and literature review | 2001 | 6 | Y | Y | Risk of seeding may be reduced by avoiding "pull" technique of PEG tube placement. |
|  | Metastatic spread to a percutaneous gastrostomy site from head and neck cancer: case report and literature review | 2005 | 5 | Y | Y | Direct tumor implantation as a result of instrumentation is the most likely explanation for the PEG tube site metastases described in this paper. |
|  | Risk factors and risk reduction of malignant seeding of the percutaneous endoscopic gastrostomy track from pharyngoesophageal malignancy: a review of all 44 known reported cases | 2007 | 5 | Y | Y | Strong risk factors for stomal metastases following PEG tube placement include pharyngoesophageal primary cancer, squamous cell histology, less well-differentiated cancer, large size, and advanced cancer stage. The risk may be reduced in patients with risk factors by radiotherapy, chemotherapy, or cancer surgery before PEG; by substituting the push-guidewire for the pull-string technique for PEG; and possibly by use of a sheath with the pull-string technique. |
|  | Percutaneous endoscopic gastrostomy site metastasis | 2007 | 6 | Y | N | This review concluded that the benefits of PEG tubes far outweigh the risk of metastasis (~0.1%). |
|  | Unexpected high rate of malignant seeding by percutaneous endoscopic gastrostomy (PEG) implantation: Final results of a prospective study? | 2012 | 4 | Y | Y | This study showed a high rate (22.5%) of direct malignant seeding in patients with esophageal and ENT cancers after “pull-through” PEG tube placement and a 16.6% long-term development of local metastases. This risk is higher in older patients, patients with higher tumor stages, and patients with squamous cell cancers, for whom direct placement should be favored. |
|  | Metastasis of untreated head and neck cancer to percutaneous gastrostomy tube exit sites | 2012 | 5 | Y | Y | PEG placement is associated with an increased risk of seeding at the PEG tube exit site. |
|  | Percutaneous endoscopic gastrostomy site metastasis from head and neck squamous cell carcinoma: case series and literature review | 2013 | 5 | Y | Y | PEG site metastatic disease portends a poor prognosis. Early detection and aggressive therapy may provide a chance of cure. Changes in PEG technique or in timing of adjunctive therapies are possible avenues in further research to prevent this complication. |
|  | Malignant Seeding Following Percutaneous Endoscopic Gastrostomy Placement in Head and Neck Cancer: Review of Literature | 2014 | 5 | Y | Equivocal | The reported rate of PEG site metastasis is 1-2% in head and neck cancer patients by the pull method. The proposed mechanism is direct tumor cell inoculation by trauma during PEG placement. There has been some retrospective studies which have shown that the push technique is better than pull through technique to prevent this complication. Randomized studies are needed to compare the complication rate between these two techniques. |
|  | Risk of esophageal cancer following percutaneous endoscopic gastrostomy in head and neck cancer patients: A nationwide population-based cohort study in Taiwan | 2016 | 4 | Y | Y | Head and neck cancer patients treated with pull-through PEG tube placement were associated with a higher risk of developing esophageal cancer, which could be fixed by surgically placed tubes. |
|  | Incidence of abdominal wall metastases following percutaneous endoscopic gastrostomy placement in patients with head and neck cancer | 2017 | 4 | Y | N | Abdominal wall metastases following PEG placement are a rare (0.64% incidence) but serious complication in patients with head and neck malignancy. While surgical technique may play a role, factors like tumor biology and overall poor patient prognosis may play a significant role in PEG site metastasis, independently of the technique used. |
|  | Risk of tumor implantation in percutaneous endoscopic gastrostomy in the upper aerodigestive tumors | 2018 | 5 | Y | N | Older patients and higher tumor stages show a greater incidence of recurrence, but no changes are explicitly recommended |
|  | Tumor implantation following percutaneous endoscopic gastrostomy insertion for head and neck and oesophageal cancer: Review of the literature | 2019 | 5 | Y | N | This study concluded that the incidence of tumor seeding after PEG tube placement was very low (<1/1000) |
| Fine needle aspiration (FNA) and Core needle biopsy (CNB) | Needle tract implantation of papillary thyroid carcinoma after fine-needle aspiration biopsy | 2005 | 4 | Y | N | FNAB remains the most useful technique for diagnosing thyroid carcinoma. The incidence of implantation was low, and when it did occur, the tumors could be surgically removed without recurrence. |
|  | A systematic review of cases reporting needle tract seeding following thyroid fine needle biopsy | 2010 | 5 | Y | N | While proper FNB technique can reduce risk, seeding is an unavoidable complication of FNB. However, the incidence of seeding is low and doesn't seem to affect prognosis because surgical removal without recurrence can be performed. |
|  | Tumour seeding after fine-needle aspiration and core biopsy of the head and neck--a systematic review | 2016 | 5 | Y | N | Risk of needle track seeding was extremely low in the head and neck. Core needle biopsy appears to have greater risk than FNAC. |
| Skull base surgery (Craniotomy, transseptal, transfacial, transpalatal interventions) | Surgical pathway seeding of clivo-cervical chordomas | 2014 | 5 | Y | N | Tumor recurrence in skull base surgery occurred at the site of a previous open transcervical approach, but no tumor seeding was noted following endoscopic endonasal resection. The endonasal approach offers a more direct surgical corridor, magnified visualization, and less violation of soft tissue surfaces where tumor implantation can occur. |
|  | Tumour seeding in the surgical pathway after resection of skull base chordoma | 2016 | 5 | Y | Y | The risk of surgical seeding should be taken into consideration when deciding on the surgical approach and the planning treatment volume for postoperative radiation therapy, and the surgical pathway should be included in follow-up studies to diagnose this peculiar treatment failure earlier. |
|  | Recurrence of Ventral Skull Base Lesions Attributed to Tumor Seeding: A Systematic Review | 2018 | 5 | Y | Y | Iatrogenic tumor seeding potential appears to be a function of tumor histology. Craniopharyngioma and chordoma appear to be especially high risk of iatrogenic seeding. |

| **Urology** | | | | | | |
| --- | --- | --- | --- | --- | --- | --- |
| **Instrumentation** | **Title** | **Year** | **LOE** | **Supports biological existence of seeding?** | **Supports clinical importance of seeding?** | **Conclusions** |
| Needle biopsy | Seeding and perineal implantation of prostatic cancer in the track of the biopsy needle: three case reports and a review of the literature | 1987 | 5 | Y | Y | There is a rate of 0.34% for perineal seeding following perineal needling of the cancerous prostate. In order to prevent perineal implantation, especially in patients who are at risk, it is suggested that a fine needle be employed to obtain prostatic tissue for biopsy and that every therapeutic effort be made. |
|  | Risk factors for perineal seeding of prostate cancer after needle biopsy | 1989 | 4 | Y | Y | This study concluded that although incidence of seeding is low after needle biopsy, tumor seeding has a poor prognosis, with all patients that presented with seeding having died 36 months after biopsy. |
|  | Needle biopsy associated tumor tracking of adenocarcinoma of the prostate | 1991 | 4 | Y | Y | Needle biopsy associated tumor tracking occurred with core (14 gauge) and biopsy gun needles (18 gauge). |
|  | Incidence of needle-tract seeding following prostate biopsy for suspected cancer: a review of the literature | 2015 | 5 | Y | N | The incidence of needle track seeding after prostate biopsy is low, but data quality in this study is low |
|  | Needle track seeding in renal mass biopsies | 2019 | 5 | Y | Y | Although limiting needle gauge and using a coaxial sheath would likely reduce incidence of seeding, incidence is still very low. Papillary tumors may be more prone to seeding needle tracts. |
|  | Tumour seeding in the tract of percutaneous renal tumour biopsy: A report on seven cases from a UK tertiary referral centre | 2019 | 6 | Y | Y | Their small series of needle tract seeding cases did not demonstrate any particular aspect of biopsy technique that has a greater risk of seeding, but imply that greater patient follow-up and weighing the risk of seeding beforehand is important going forward. |
|  | Needle tract seeding in renal tumor biopsies: experience from a single institution | 2021 | 4 | Y | Y | Percutaneous needle biopsy of renal masses carries a significant risk of inducing needle tract seeding in patients with renal cell carcinoma. |
| Laparoscopy | Tumor seeding in urological laparoscopy: an international survey | 2004 | 4 | Y | N | This study found a very low rate of seeding (0.1%) and concluded that laparoscopic surgery does not have a greater risk of seeding than open surgery. |
|  | Port site metastasis and tumor seeding in oncologic laparoscopic urology | 2008 | 5 | Y | Y | Tumor seeding and port site metastasis remain a valid concern during laparoscopic procedures for urologic malignancies. |
|  | Port site metastases | 2008 | 5 | Y | N | Incidence of port-site metastases appears similar to that seen after open surgery. Tumor aggressiveness appears to be a significant factor in likelihood of seeding. |
|  | Laparoscopic port-site metastasis in urologic surgery | 2008 | 5 | Y | N | The incidence of port-site metastases is comparable with that seen following open surgery. The etiology is likely multifactorial, but adhering to proper surgical technique is the most effective preventative strategy. |
|  | Port site metastasis after surgery for renal cell carcinoma: Harbinger of future metastasis | 2014 | 5 | Y | N | Port site metastasis for renal cell carcinoma is rare and carries a poor prognosis. In most cases, it is not an isolated metastasis but rather a harbinger of progressive disease. Biological factors like higher tumor grade contribute to port site metastasis. |
| Ureteroscopy | Diagnostic Ureterorenoscopy Is Associated with Increased Intravesical Recurrence following Radical Nephroureterectomy in Upper Tract Urothelial Carcinoma | 2015 | 4 | Y | Y | Diagnostic ureterorenoscopy for upper tract urothelial carcinoma is associated with an increased intravesical recurrence rate after radical nephroureterectomy. |
|  | Diagnostic Ureteroscopy Prior to Radical Nephroureterectomy for Upper Tract Urothelial Carcinoma Increased the Risk of Intravesical Recurrence | 2018 | 5 | Y | Y | This study concluded that diagnostic ureteroscopy increases the intravesicular recurrence risk in patients with UTUC and recommend developing methods of chemoprophylaxis after endoscopy to prevent recurrence. |
|  | The Impact of Upper Tract Urothelial Carcinoma Diagnostic Modality on Intravesical Recurrence after Radical Nephroureterectomy: A Single Institution Series and Updated Meta-Analysis | 2021 | 4 | Y | Y | This study concluded that ureteroscopy increased incidence of intravesicular recurrence of urothelial carcinoma. |
|  | Risk Factors for Intravesical Recurrence after Minimally Invasive Nephroureterectomy for Upper Tract Urothelial Cancer (ROBUUST Collaboration) | 2021 | 4 | Y | Y | Intravesical recurrence after minimally invasive radial nephroureterectomy for upper tract urothelial carcinoma is a relatively common event. Risk factors include a ureteroscopic biopsy, transurethral resection of the bladder cuff, and positive surgical margins. |

| **Gynecologic Oncology** | | | | | | |
| --- | --- | --- | --- | --- | --- | --- |
| **Instrumentation** | **Title** | **Year** | **LOE** | **Supports biological existence of seeding?** | **Supports clinical importance of seeding?** | **Conclusions** |
| Laparoscopy | Port site recurrences after laparoscopic and thoracoscopic procedures in malignancy | 1996 | 5 | Y | Equivocal | Until valid prospective data on PSR frequency are available, laparoscopic or thoracoscopic resection of malignancy off-protocol should be undertaken with circumspection. |
|  | Risk factors contributing to early occurrence of port site metastases of laparoscopic surgery for malignancy | 1999 | 5 | Y | Y | Most port site metastases (PSM) were adenocarcinomas, advanced stage, and with diffuse peritoneal carcinomatosis. Risk factors for early PSM were ovarian cancers, ascites, and diagnostic/palliative procedures for malignancy. Practitioners should exercise caution when using laparoscopic techniques in these patients. |
|  | Port-site metastasis after laparoscopic surgery for gynecologic cancer. A report of six cases | 2000 | 6 | Y | Y | Patients who have a malignant ovarian tumor and ascites should not be treated with laparoscopic surgery due to risk of seeding. |
|  | Port site metastases after laparoscopic lymph node staging of cervical carcinoma | 2001 | 6 | Y | Y | The frequency of port site metastasis might be higher than expected. Therefore, surgeons should reduce mechanical irritation of port sites and spillage of tumor cells. |
|  | Subcutaneous tumor implantation after laparoscopic procedures in women with malignant disease | 2004 | 4 | Y | Y | Laparoscopy-related subcutaneous tumor implantation is rare (0.97%) in women undergoing transperitoneal laparoscopy with malignant disease. Subcutaneous implantation appears to occur in patients with known metastatic disease alongside synchronous advanced intraabdominal or pelvic metastasis and progression of carcinomatosis. |
|  | Laparoscopic port-site metastases in patients with gynecological malignancies | 2004 | 5 | Y | Y | Laparoscopic port-site metastases are a potential complication of laparoscopy in patients with gynecological malignancies, even in patients with early-stage disease. |
|  | Low incidence of port-site metastases after laparoscopic staging of uterine cancer | 2010 | 4 | Y | N | PSM is a rare complication of laparoscopic staging for endometrial and cervical cancer with no predictive factors. Isolated PSM can be virtually eliminated with adequate operative technique. PSM in patients with uterine cancer cannot be used as an argument against laparoscopic staging in uterine cancer. |
|  | Iatrogenic transtubal spill of endometrial cancer: risk or myth** | 2011 | 5 | Y | N | Investigation into the rate of spill of EC cells during laparoscopic surgery is in the early stages and not yet definitive. |
|  | Port-site metastasis after laparoscopic surgical staging of endometrial cancer: a systematic review of the published and unpublished data | 2012 | 5 | Equivocal | N | Five patients had isolated port metastases, while the remaining patients had poly-metastasis. The study argues that there is not enough data to conclude that laparoscopy presents a risk for iatrogenic tumor seeding, and maybe be a "hidden, disseminated, micro-metastatic disease." |
|  | Port site metastases after robot-assisted surgery: a systematic review | 2013 | 5 | Y | Y | Port-site metastasis is an extremely rare complication of robotic surgery. No safe conclusion can be drawn, but the aim of this study was to raise doctors' suspicion levels to such a rare new entity. |
| Hysteroscopy | Does hysteroscopy facilitate tumor cell dissemination? Incidence of peritoneal cytology from patients with early stage endometrial carcinoma following dilatation and curettage (D & C) versus hysteroscopy and D & C | 2000 | 4 | Y | Y | This study concluded that hysteroscopic tumor cell dissemination of carcinoma cells via fluid hysteroscopy occurred significantly more often for patients who underwent hysteroscopy before surgical staging than for those who did not undergo hysteroscopy. |
|  | Iatrogenic transtubal spill of endometrial cancer: risk or myth** | 2011 | 5 | Y | Equivocal | The majority of the studies about EC cell dissemination during H/S and SIS suggest that they increase the risk of spill. There is insufficient data to comment definitively on the viability of the disseminated EC cells. |
| Morcellation | Impact of morcellation on survival outcomes of patients with unexpected uterine leiomyosarcoma: A systematic review and meta-analysis. | 2015 | 5 | Y | Y | This data supports a significant correlation between uterine morcellation and an increased risk of intra-abdominal recurrence in patients affected by unexpected uterine leiomyosarcoma. Further studies are needed to estimate a risk to benefit ratio of morcellation in patients with uterine fibroids and undiagnosed ULMS. |
|  | Peritoneal dissemination complicating morcellation of uterine mesenchymal neoplasms. | 2012 | 4 | Y | Y | While additional study is warranted, these data suggest uterine morcellation carries a risk of disseminating unexpected malignancy with apparent associated increase in mortality much higher than appreciated currently. |
| Episiotomy | The implantation of cervical neoplasia at postpartum episiotomy scar: the clinical evidence | 2015 | 5 | Y | Y | Clinicians should carefully examine not only the cervix at the time of labor, but also the episiotomy scar in women following a pregnancy complicated by cervical cancer, given these 18 patients with cervical cancer recurrence in episiotomy scars after vaginal deliveries. |
| Saline Infusion Sonohysterogram (SIS) | Iatrogenic transtubal spill of endometrial cancer: risk or myth** | 2011 | 5 | Y | Equivocal | The majority of the studies about EC cell dissemination during H/S and SIS suggest that they increase the risk of spill. There is insufficient data to comment definitively on the viability of the disseminated EC cells. |
| Dilation and Curettage (D&C) | Iatrogenic transtubal spill of endometrial cancer: risk or myth** | 2011 | 5 | Y | N | Investigation into the rate of spill of EC cells during D&C is in the early stages and not yet definitive. |

| **Orthopedic Surgery** | | | | | | |
| --- | --- | --- | --- | --- | --- | --- |
| **Instrumentation** | **Title** | **Year** | **LOE** | **Supports biological existence of seeding?** | **Supports clinical importance of seeding?** | **Conclusions** |
| Open biopsy | Are Biopsy Tracts a Concern for Seeding and Local Recurrence in Sarcomas?** | 2017 | 4 | Y | Y | Open biopsies were associated with an increased risk of tumoral seeding of the biopsy site, and tumoral seeding was associated with an increased risk of local recurrence. |
| Percutaneous needle biopsy | Needle tract seeding after percutaneous biopsy of sarcoma: Risk/benefit considerations | 2017 | 5 | Y | N | The benefits of pretreatment biopsy in patients with sarcomas (extremity, retroperitoneal, intraperitoneal) outweigh the potential risks of needle tract seeding (0.37%). |
|  | Are Biopsy Tracts a Concern for Seeding and Local Recurrence in Sarcomas?** | 2017 | 4 | Y | Y | Open biopsies were associated with an increased risk of tumoral seeding of the biopsy site, and tumoral seeding was associated with an increased risk of local recurrence. |

| **Dermatology** | | | | | | |
| --- | --- | --- | --- | --- | --- | --- |
| **Instrumentation** | **Title** | **Year** | **LOE** | **Supports biological existence of seeding?** | **Supports clinical importance of seeding?** | **Conclusions** |
| Percutaneous needle biopsy | Does needle biopsy cause an increased risk of extracapsular extension in the diagnosis of metastatic lymph node in melanoma? | 2018 | 4 | Y | Y | The proportion of extracapsular extension was significantly greater in the needle biopsy group (28/37) than in patients who underwent surgical adenectomy. Our results suggest an increased risk of extracapsular extension after external lymph node biopsy in cases of suspicion of metastatic lymph node of melanoma. This encourages us to prefer surgical adenectomy in patients with suspected adenopathy. |

| **Ophthalmology** | | | | | | |
| --- | --- | --- | --- | --- | --- | --- |
| **Instrumentation** | **Title** | **Year** | **LOE** | **Supports biological existence of seeding?** | **Supports clinical importance of seeding?** | **Conclusions** |
| Fine needle aspiration (FNA) | Quantitation of tumor seeding from fine needle aspiration of ocular melanomas | 1988 | 6 | Y | N | While 14 of 21 (67%) of all fine needle aspiration tracts and eight of 15 (53%) of indirect tracts contained tumor cells, the number of tumor cells was less than that associated with tumor growth in experimental models. Indirect aspirate tracts contained significantly fewer cells than tracts of direct aspirates (P less than .001). |

| **General Surgery** | | | | | | |
| --- | --- | --- | --- | --- | --- | --- |
| **Instrumentation** | **Title** | **Year** | **LOE** | **Supports biological existence of seeding?** | **Supports clinical importance of seeding?** | **Conclusions** |
| Needle Biopsy | Implantation metastases from gastrointestinal cancer after percutaneous puncture or biliary drainage | 1996 | 6 | Y | Y | Study recommends that fine-needle biopsy should be restricted to patients who will benefit from a more accurate preoperative diagnosis. |
|  | Are malignant cells displaced by large-gauge needle core biopsy of the breast? | 1999 | 4 | Y | N | Tumor cell displacement was observed in 32% of patients who had undergone large-gauge needle core biopsy. The incidence and amount of tumor displacement was inversely related to the interval between core biopsy and excision. This relation suggests that tumor cells do not survive displacement. |
|  | Needle-tract implantation in hepatocellular carcinoma: frequency and CT findings after biopsy with a 19.5-gauge automated biopsy gun | 2000 | 4 | Y | Equivocal | The frequency of needle-tract implantation of HCC after percutaneous needle biopsy was higher than reported previously, and careful attention should be paid during interpretation of CT images in patients with a history of previous percutaneous biopsy. |
|  | Risk of needle-track seeding after diagnostic image-guided core needle biopsy in breast cancer. | 2002 | 4 | Y | N | Did not find an increased rate of recurrence due to needle-track seeding, and IGCNB remains our procedure of choice for diagnosing mammographically detected suspicious breast lesions. |
|  | Risk of dissemination with biopsy of colorectal liver metastases | 2003 | 4 | Y | N | There is a significant risk of local dissemination with biopsy of colorectal liver metastases but this risk is independent of biopsy type. |
|  | Needle tract implantation of hepatocellular carcinoma and pancreatic carcinoma after ultrasound-guided percutaneous puncture: clinical and pathologic characteristics and the treatment of needle tract implantation | 2004 | 3 | Y | Y | These results suggest that needle tract implantation develops regardless of the procedure or the pathologic differentiation of the primary tumor, and that surgical resection might be effective for controlling these implanted lesions. |
|  | Tumour cell displacement after 14G breast biopsy | 2004 | 4 | Y | N | Do not recommend changes - excision of needle tracts is not feasible and unadvised since radiotherapy is recommended after needle biopsy. |
|  | Biopsy of resectable colorectal liver metastases causes tumour dissemination and adversely affects survival after liver resection | 2005 | 4 | Y | Y | Yes - needle biopsy should not be performed if a curative option is available. |
|  | Risk of tumour seeding after percutaneous radiofrequency ablation for hepatocellular carcinoma | 2005 | 4 | Y | Y | Recommend avoiding RFA if a curative treatment (i.e. liver transplant) is a possibility in the future. |
|  | Needle tract seeding after radiofrequency ablation of hepatic tumors | 2005 | 4 | Y | Y | The results show that the frequency of this complication is not insignificant, and are at the upper end of rates reported in the literature of 0.5% to 2.8%. Specific risk factors identified in this study include treatment of subcapsular lesions, patients treated in multiple sessions, and lesions requiring more than one electrode placement. |
|  | Needle tract implantation after sonographically guided percutaneous biopsy of hepatocellular carcinoma: evaluation of doubling time, frequency, and features on CT | 2005 | 4 | Y | N | Needle tract implantation was rare. The doubling times of extrahepatic needle tract implantation of malignant neoplasms after sonographically guided percutaneous biopsy for HCC were similar to those of typical HCCs in the liver on CT-based analysis. |
|  | Epithelial displacement during breast needle core biopsy causes diagnostic difficulties in subsequent surgical excision specimens | 2006 | 6 | Y | Y | Previous observations that epithelial displacement is more likely to occur when the interval between NCB and surgical excision is short are supported. |
|  | Needle tract implantation of hepatocellular carcinoma after fine needle biopsy | 2007 | 6 | Y | Y | This study supports a policy of selective use of fine needle biopsy for the definitive diagnosis of liver lesions |
|  | Risk of needle tract seeding of breast cancer: cytological results derived from core wash material | 2008 | 4 | Y | Equivocal | In conclusion, the incidence of positive cases of cytology derived from ultrasonographically guided breast core needles' wash material was 65%. The clinical significance is debatable; however, there may be a theoretical risk of local recurrence if the tract is not excised or radiotherapy not given. |
|  | Breast cancer seeding associated with core needle biopsies: a systematic review | 2009 | 5 | Y | N | Although data are limited, no increased morbidity has been associated with iatrogenic seeding after CNB. |
|  | The use of positive core wash cytology to estimate potential risk of needle tract seeding of breast cancer: directional vacuum-assisted biopsy versus automated core needle biopsy** | 2010 | 4 | Y | Y | The use of the directional vacuum-assisted device significantly decreases the potential risk of needle tract seeding of breast cancer after an ultrasonographically guided needle biopsy. |
|  | Treatment and clinical outcome of needle-track seeding from hepatocellular carcinoma | 2011 | 4 | Y | Y | Suggest that en-bloc excision is optimal treatment paradigm for HCC as there is no risk of needle track seeding. |
|  | Trans-peritoneal fine needle aspiration biopsy of hilar cholangiocarcinoma is associated with disease dissemination | 2011 | 4 | Y | Y | FNA should not be performed if a curative option is available. |
|  | Risk of peritoneal carcinomatosis by endoscopic ultrasound-guided fine needle aspiration for pancreatic cancer | 2013 | 4 | Y | N | EUS-FNA for pancreatic cancer did not significantly increase the risk of peritoneal carcinomatosis. |
|  | Peritoneal seeding in intraductal papillary mucinous neoplasm of the pancreas patients who underwent endoscopic ultrasound-guided fine-needle aspiration: the PIPE Study | 2014 | 4 | Y | N | In this cohort of patients undergoing resection of IPMN, the difference in the frequency of peritoneal seeding in the EUS-FNA group and the No Sampling group was not significant. |
|  | Seeding after ultrasound-guided percutaneous biopsy of liver metastases in patients with colorectal or breast cancer | 2016 | 4 | Y | N | The results showed no seeding in breast cancer patients. Seeding rate after biopsy in colorectal cancer patients is not negligible, however, without affecting outcome. |
|  | Breast cancer neoplastic seeding in the setting of image-guided needle biopsies of the breast | 2017 | 4 | Y | Y | High-grade, triple-negative breast cancers and multiple-insertion, non-coaxial biopsies may be risk factors for NS. NS should be suspected on the basis of the superficial and linear pattern of disease progression in these patients. |
|  | Needle tract seeding following core biopsies in retroperitoneal sarcoma | 2017 | 4 | Y | N | The risk of a needle tract metastasis after core needle biopsy for retroperitoneal sarcoma is very low but not zero. The safest method seems a trans-retroperitoneal approach with a co-axial technique. Local recurrence rate is not altered after doing a core needle biopsy. |
|  | Tumor cell seeding in the biopsy tract and its clinical significance in osteosarcomas | 2018 | 4 | Y | Y | This study suggests that the biopsy tract must also be resected to prevent metastasis after CNB. |
|  | Preoperative EUS-guided FNA: effects on peritoneal recurrence and survival in patients with pancreatic cancer | 2018 | 4 | Y | N | Preoperative EUS-FNA for pancreatic cancer was not associated with an increased rate of peritoneal recurrence or mortality. Therefore, EUS-FNA is an accurate and safe method to obtain suspicious pancreatic mass tissue. |
|  | Comparison of a coaxial versus non-coaxial liver biopsy technique in an oncological setting: diagnostic yield, complications and seeding risk | 2020 | 4 | Y | Y | Co-axial liver biopsy had better diagnostic results and lower risk of seeding than non-coaxial. |
|  | Overlooked risk for needle tract seeding following endoscopic ultrasound-guided minimally invasive tissue acquisition | 2020 | 5 | Y | Y | Recommend shortening the puncture path, limiting the number of puncture, paying attention to the procedure method, and adding seeding needle sleeve to reduce risk of seeding |
|  | Preoperative Endoscopic Ultrasound Fine Needle Aspiration Versus Upfront Surgery in Resectable Pancreatic Cancer: A Systematic Review and Meta-analysis of Clinical Outcomes Including Survival and Risk of Tumor Recurrence | 2021 | 1 | Y | Equivocal | Preoperative EUS-FNA in resectable pancreatic cancer may be associated with significantly greater OS when compared to the non-FNA group, with no significant difference in the rates of tumor recurrence or peritoneal seeding. |
|  | Analysis of circulating cell-free DNA after endoscopic ultrasound-guided fine needle aspiration in pancreatic ductal adenocarcinoma | 2021 | 6 | Y | N | The increase in cfDNA concentration after EUS-FNA was not caused by tumor cell components released into blood vessels. Hence, the risk of seeding via the blood stream after EUS-FNA may need not be considered. |
|  | Needle tract seeding after endoscopic ultrasound-guided tissue acquisition of pancreatic tumors: Nationwide survey in Japan | 2022 | 4 | Y | Y | NTS appeared only after transgastric not after transduodenal EUS-TA. Careful follow-up provides an opportunity to remove localized NTS lesions by gastrectomy. |
| Catheter Procedures | Subcutaneous metastasis of pancreatic cancer in the site of percutaneous biliary drainage | 2003 | 5 | Y | Y | This study suggests that seeding can be avoided if patients at risk, whenever possible, undergo endoscopic drainage. |
|  | Needle track seeding in hepatocellular carcinoma after local ablation by high-dose-rate brachytherapy: a retrospective study of 588 catheter placements | 2018 | 4 | Y | Y | To compensate for the risk of seeding, a track irradiation technique similar to track ablation in RFA should be implemented in clinical routine |
| Laparoscopy | Abdominal wall metastases following laparoscopy | 1994 | 5 | Y | Y | First, there may be increased exfoliation of tumour cells following manipulation by laparoscopic instruments of an unsuspected malignancy. Second, there may be repeated close contact between tumour-laden instruments and the port. Third, the passage of resected tissue through a small incision may coat the wound with potentially malignant cells. |
|  | Port site metastasis. An unresolved problem in laparoscopic surgery. A review | 1995 | 5 | Y | Y | This study suggests that prophylactic measures should be used to reduce risk (including protective bags, peritoneal lavage with heparin, cytocidal solutions) |
|  | Wound recurrence following conventional treatment of colorectal cancer. A rare but perhaps underestimated problem | 1996 | 4 | Y | Y | Incisional recurrence is likely underestimated, although uncommon, and is likely a harbinger of diffuse intra-abdominal disease. |
|  | Port site recurrences after laparoscopic and thoracoscopic procedures in malignancy | 1996 | 5 | Y | N | Until valid prospective data on PSR frequency are available, laparoscopic or thoracoscopic resection of malignancy off-protocol should be undertaken with circumspection. |
|  | Port-site metastases in patients undergoing laparoscopy for gastrointestinal malignancy | 1996 | 4 | Y | N | Five of 20 patients with tumour involving serosal surfaces developed port-site recurrence compared with none of 26 without serosal involvement (P = 0.022, Fisher's exact test). Port-site recurrence may be related to serosal involvement with tumour. |
|  | Port site recurrences after laparoscopic surgery. A review | 1998 | 5 | Y | Y | Clinical evidence that laparoscopy with CO2 pneumoperitoneum can enhance tumor dissemination is given. Port site metastases seem to be secondary to multiple factors including the gas used, local trauma, tumor manipulation, biologic properties of the tumor, and individual surgical skills. |
|  | Risk factors contributing to early occurrence of port site metastases of laparoscopic surgery for malignancy | 1999 | 5 | Y | Y | The majority of recurrences were in patients with adenocarcinoma cell type, advanced stage (far-advanced disease), and often with diffuse peritoneal carcinomatosis, suggesting that port site metastases may contribute to the highly aggressive nature of the disease. |
|  | Tumor seeding following laparoscopy: international survey | 1999 | 4 | Y | N | Acknowledge greater risk of seeding, but do not explicitly recommend changes. |
|  | Recurrent gallbladder carcinoma along laparoscopic cholecystectomy port tracks: CT demonstration | 1999 | 6 | Y | Equivocal | Tumor recurrence along port tracks is a potential complication of laparoscopic cholecystectomy when gallbladder carcinoma is present, even after subsequent hepatic resection is performed for attempted cure. Recurrences appear as a new or enlarging abdominal wall mass, often involving subjacent omental fat, and may be the only site of recurrent disease at CT. |
|  | Trocar site recurrence in laparoscopic surgery for colorectal cancer: myth or real concern? | 2001 | 5 | Y | N | Although an overall wound recurrence rate of 0.62% is not negligible, it may be comparable to incidences of PSM across other malignancies and to incidences across other means of resection (laparotomy vs. laparoscopy). |
|  | Port site metastases: where are we at the beginning of the 21st century? | 2002 | 5 | Y | Y | Study suggests that all trocar sites should be excised and a proper open procedure conducted according to the pathological findings |
|  | The theories and realities of port-site metastases: a critical appraisal. | 2002 | 5 | Y | N | Port site metastases is not as common as first thought, and the true incidence might be comparable to open operation. The reality may be that poor surgical technique, such as improper handling of the tumor, is the cause of seeding. |
|  | PET/CT detects abdominal wall and port site metastases of colorectal carcinoma** | 2006 | 4 | Y | N | A total of 16 abdominal wall lesions were detected, located to surgical scars, stomas, drain and laparoscope ports. No recommendation for changes in practice. |
|  | The rate of port-site metastases after 2251 laparoscopic procedures in women with underlying malignant disease | 2008 | 4 | Y | N | The rate of port-site tumor implantation after laparoscopic procedures in women with malignant disease is low and almost always occurs in the setting of synchronous, advanced intraabdominal or distant metastatic disease. The presence of port-site implantation is a surrogate for advanced disease and should not be used as an argument against laparoscopic surgery in gynecologic malignancies. |
|  | Influence of laparoscopic gastrectomy on the detection rate of free gastric cancer cells in the peritoneal cavity | 2010 | 4 | Y | N | Laparoscopic technique did not increase risk compared to open technique. |
|  | Predicting factors of unexpected peritoneal seeding in locally advanced gastric cancer: indications for staging laparoscopy | 2010 | 4 | Y | N | Tumor type (Borrmann type 3 or 4), tumor invasion, tumor size, and tumors involving the anterior wall were predictive factors for distant peritoneal seeding. |
|  | Exfoliated Tumor Cells in Intraluminal Lavage Samples after Colorectal Endoscopic Submucosal Dissection: A Pilot Study | 2014 | 6 | Y | Y | Sufficient intraluminal lavage after colorectal endoscopic submucosal dissection is necessary to remove exfoliated tumor cells |
|  | Colonic perforation either during or after stent insertion as a bridge to surgery for malignant colorectal obstruction increases the risk of peritoneal seeding | 2015 | 4 | Y | Y | Colorectal stenting as a bridge to surgery showed no significant short- or long-term benefits compared with surgery only, and was associated with peritoneal seeding after perforation. Stenting before surgery should therefore only be considered in patients with a high risk of complications associated with emergency surgery. |
|  | Port-site metastasis after laparoscopic surgery for gastrointestinal cancer | 2017 | 1 | Y | N | Do not explicitly recommend changes - "technical aspects may be the biggest cause of PSM, and accordingly, surgeons should make a maximum effort to prevent PSM" |
|  | Tumor Seeding During Colonoscopy as a Possible Cause for Metachronous Colorectal Cancer | 2019 | 6 | Y | Equivocal | Although our study does not eliminate other possibilities of transmission, our findings and experiments support the hypothesis that tumor seeding can occur during colonoscopy via the working channel of the endoscope. The possibility of iatrogenic seeding seems low. |
|  | Extent of intraluminal exfoliated malignant cells during surgery for colon cancer: Differences in cell abundance ratio between laparoscopic and open surgery** | 2019 | 4 | Y | Y | Exfoliated malignant cells were detected at anastomosis sites in patients with colon cancer. On the distal side, laparoscopic colectomy may prevent the development of exfoliated malignant cells. |
|  | Surgical Treatment of Port-Site Metastases After Laparoscopic Radical Resection of Gastrointestinal Tumors | 2020 | 4 | Y | N | Reports on 8 cases of port-site metastasis without any proposed clinical changes. |
|  | Foreign body reaction mimicking local recurrence from polyactide adhesion barrier film after laparoscopic colorectal cancer surgery: A retrospective cohort study | 2022 | 4 | Y | Y | This study suggested that the placement of PLA adhesion barrier should be avoided after laparoscopic procedure. |
| Probe procedures | Increased detection rate and potential prognostic impact of disseminated tumor cells in patients undergoing endorectal ultrasound for rectal cancer | 2007 | 4 | Y | Y | This study demonstrates significantly increased hematogenous tumor-cell dissemination after endorectal ultrasound in rectal cancer patients. Patients with tumor cells in blood samples after endorectal ultrasound tend to have a worse prognosis. |
|  | Neoplastic Seeding After Radiofrequency Ablation for Hepatocellular Carcinoma | 2008 | 4 | Y | Y | Indication for RFA should be carefully considered for HCC patients with larger or more poorly differentiated tumors |
|  | The use of positive core wash cytology to estimate potential risk of needle tract seeding of breast cancer: directional vacuum-assisted biopsy versus automated core needle biopsy** | 2010 | 4 | Y | Y | The use of the directional vacuum-assisted device significantly decreases the potential risk of needle tract seeding of breast cancer after an ultrasonographically guided needle biopsy. |
| Open surgery | Tumor recurrence in the abdominal wall scar tissue after large-bowel cancer surgery. | 1983 | 6 | Y | N | Data suggests incisional recurrence is a manifestation of disseminated cancer rather than isolated implantation. |
|  | Does seeding of intraluminal tumour cells cause anastomotic recurrence after oesophageal resection? | 2002 | 6 | Y | Y | This study suggests that the gastric tube reconstruction should be washed thoroughly with a cytotoxic agent to reduce the risk of metastasis. |
|  | Disseminated single tumor cells as detected by real-time quantitative polymerase chain reaction represent a prognostic factor in patients undergoing surgery for colorectal cancer | 2002 | 3 | Y | Y | Identification of patients at high risk for metastatic disease after curative resection of colorectal cancer might be improved by analyzing peritoneal lavage specimens |
|  | PET/CT detects abdominal wall and port site metastases of colorectal carcinoma** | 2006 | 4 | Y | N | A total of 16 abdominal wall lesions were detected, located to surgical scars, stomas, drain and laparoscope ports. No recommendation for changes in practice. |
|  | Extent of intraluminal exfoliated malignant cells during surgery for colon cancer: Differences in cell abundance ratio between laparoscopic and open surgery** | 2019 | 4 | Y | Y | Exfoliated malignant cells were detected at anastomosis sites in patients with colon cancer. On the distal side, laparoscopic colectomy may prevent the development of exfoliated malignant cells. |

| **Gastroenterology** | | | | | | |
| --- | --- | --- | --- | --- | --- | --- |
| **Instrumentation** | **Title** | **Year** | **LOE** | **Supports biological existence of seeding?** | **Supports clinical importance of seeding?** | **Conclusions** |
| Needle Procedures | CT of abdominal wall implantation metastases after abdominal percutaneous procedures | 1998 | 4 | Y | N | In most cases of abdominal wall implantation metastasis following abdominal percutaneous procedure, CT shows additional intraabdominal tumor sites. This complication may occur following a variety of abdominal percutaneous procedures (either radiological or surgical). |
|  | Seeding Risk Following Percutaneous Approach to Hepatocellular Carcinoma - Seeding following percutaneous diagnostic and therapeutic approaches for hepatocellular carcinoma. What is the risk and the outcome? Seeding risk for percutaneous approach of HCC | 2007 | 6 | Y | Y | Study concluded that risk of seeding in hepatocellular carcinoma appears greater when using a purely diagnostic biopsy compared to therapeutic percutaneous procedures. |
|  | Incidence of needle tract seeding and responses of soft tissue metastasis by hepatocellular carcinoma postradiotherapy | 2007 | 4 | Y | N | Patients whose metastatic lesions were considered procedure-related (PR) i.e. due to needle tract seeding had better prognoses than those with non-procedure-related (NPR) metastasis. Reinforces needle tract seeding as a complication of needle biopsy with no recommended changes. |
|  | Analysis of patients with tumor seeding after percutaneous radiofrequency ablation of hepatocellular carcinoma | 2008 | 4 | Y | Y | The selection of the proper RFA system to avoid multiple sessions and the use of ablation technique are important for the prevention of seeding. Additionally, long-term follow-up after RFA by extensive imaging of the pelvic cavity and the thoracoabdominal wall is needed. |
|  | Needle track seeding following biopsy of liver lesions in the diagnosis of hepatocellular cancer: a systematic review and meta-analysis | 2008 | 5 | Y | N | In this systematic review we have shown that the incidence of needle tract tumour seeding following biopsy of a HCC is 2.7% overall, or 0.9% per year. |
|  | Needle track seeding after percutaneous microwave ablation of malignant liver tumors under ultrasound guidance: analysis of 14-year experience with 1462 patients at a single center | 2011 | 4 | Y | N | The results showed that the neoplastic seeding was a low risk complication of percutaneous MWA of liver cancer and was considered acceptable in general. |
|  | Tumour seeding after percutaneous cryoablation for hepatocellular carcinoma | 2012 | 4 | Y | Y | The risk of seeding after cryoablation for HCC is small. Direct puncture of subcapsular tumours should be avoided to minimize seeding. |
|  | Risk of gastric or peritoneal recurrence, and long-term outcomes, following pancreatic cancer resection with preoperative endosonographically guided fine needle aspiration | 2013 | 4 | Y | N | Pre-operative EUS-FNA was not associated with an increased rate of gastric or peritoneal cancer recurrence in patients with resected pancreatic cancer. |
|  | Percutaneous Preoperative Biliary Drainage for Resectable Perihilar Cholangiocarcinoma: No Association with Survival and No Increase in Seeding Metastases | 2015 | 4 | Y | N | The present study found no effect of PTBD on survival compared to patients with EBD and no increase in seeding metastases that developed as initial recurrence. These data suggest that PTBD can safely be used in preoperative management of PHC. |
|  | Needle Tract Seeding: An Overlooked Rare Complication of Endoscopic Ultrasound-Guided Fine-Needle Aspiration | 2017 | 5 | Y | Y | Careful follow-up using endoscopy and imaging modalities is necessary in cases in which the needle tract site is outside the surgical resection margins |
|  | Rate of seeding with biopsies and ablations of hepatocellular carcinoma: A retrospective cohort study | 2017 | 4 | Y | Equivocal | Biopsies of liver masses are associated with a low rate of wall seeding when performed in a community setting and when they are the sole procedures. Ablations may have a higher rate of seeding, particularly if done with biopsies, but are still rare. |
|  | Needle track seeding after percutaneous radiofrequency ablation of hepatocellular carcinoma: 14-year experience at a single centre | 2017 | 4 | Y | N | Study describes risk factors such size of the tumor (>3cm) but do not explicitly recommend changes. |
|  | A systematic review of the comparison of the incidence of seeding metastasis between endoscopic biliary drainage and percutaneous transhepatic biliary drainage for resectable malignant biliary obstruction | 2019 | 5 | Y | Y | This study concluded that endoscopic biliary drainage had a much lower risk of seeding than percutaneous transhepatic biliary drainage in patients with cholangiocarcinoma. |
|  | Non-negligible rate of needle tract seeding after endoscopic ultrasound-guided fine-needle aspiration for patients undergoing distal pancreatectomy for pancreatic cancer | 2020 | 4 | Y | N | Although preoperative EUS-FNA for pancreatic body and tail cancer has no negative effect on recurrence-free surgical or overall survival, needle tract seeding after EUS-FNA was observed to have a non-negligible rate. |
| Catheter Procedures | Percutaneous transhepatic biliary drainage catheter tract recurrence in cholangiocarcinoma | 2010 | 4 | Y | Y | PTBD catheter tract recurrence is not unusual. The prognosis for these patients is generally poor, even after resection. To prevent this troublesome complication, endoscopic biliary drainage is first recommended when drainage is indicated. |
|  | Needle track seeding in colorectal carcinoma after local ablation by high-dose-rate brachytherapy: a retrospective study of 1,107 catheter placements | 2022 | 4 | Y | N | Brachytherapy for treatment of colorectal metastases is associated with a similar risk for extra-hepatic track seeding compared to radio-frequency ablation (RFA). |
| Laparoscopy | Incidence of port site recurrence after laparoscopic cholecystectomy for preoperatively unsuspected gallbladder carcinoma | 1998 | 4 | Y | N | Do not explicitly recommend changes, but suggest that perforation of gallbladder in patients with undiagnosed adenocarcinoma greatly increases risk of seeding. |
|  | Port site metastases from gallbladder cancer after laparoscopic cholecystectomy. Results of a Swedish survey and review of published reports | 1999 | 4 | Y | Y | Port site metastases from gallbladder cancer may be more common than previously thought. A laparoscopic procedure should not be done if cancer of the gallbladder is suspected. |
|  | Port-site metastasis following laparoscopic cholecystectomy: a review of the literature and a case report | 2000 | 6 | Y | Y | The study suggests that if malignancy is detected during laparoscopy early conversion to open procedure is recommended. |
|  | Port site recurrences after laparoscopic cholecystectomy | 2001 | 5 | Y | Y | Study concluded that long-term prognosis of patients with cancer unknown prior to biopsy is worsened by laparoscopic intervention, and should therefore be avoided when curative options are available |
|  | Port site metastasis after diagnostic laparoscopy for upper gastrointestinal tract malignancies: an uncommon entity | 2002 | 4 | Y | N | Port site implantation after diagnostic laparoscopy for upper GI malignancy is uncommon, does not seem to be different from open incision site recurrence, and occurs in the setting of advanced disease. Therefore, the risk of port site recurrence cannot be used as an argument against laparoscopy in upper GI malignancy. |
|  | Dissemination metastasis after laparoscopic colorectal surgery versus conventional open surgery for colorectal cancer: a metanalysis | 2013 | 5 | Y | N | No significant differences found between laparoscopy and open surgery |
